# Strategies that facilitate the delivery of exceptionally good patient care in general practice: a qualitative study with patients and primary care professionals

**DOI:** 10.1186/s12875-024-02352-1

**Published:** 2024-04-27

**Authors:** Roisin O’Malley, Paul O’Connor, Sinéad Lydon

**Affiliations:** Discipline of General Practice, University of Galway, Newcastle, 1 Distillery Road, Galway, H91TK33 Ireland

**Keywords:** Positive deviance, Safety-II, Quality improvement, General practice, Primary care

## Abstract

**Background:**

In recent years, proactive strengths-based approaches to improving quality of care have been advocated. The positive deviance approach seeks to identify and learn from those who perform exceptionally well. Central to this approach is the identification of the specific strategies, behaviours, tools and contextual strategies used by those positive deviants to perform exceptionally well. This study aimed to: identify and collate the specific strategies, behaviours, processes and tools used to support the delivery of exceptionally good care in general practice; and to abstract the identified strategies into an existing framework pertaining to excellence in general practice; the Identifying and Disseminating the Exceptional to Achieve Learning (IDEAL) framework.

**Methods:**

This study comprised a secondary analysis of data collected during semi-structured interviews with 33 purposively sampled patients, general practitioners, practice nurses, and practice managers. Discussions explored the key factors and strategies that support the delivery of exceptional care across five levels of the primary care system; the patient, provider, team, practice, and external environment. For analysis, a summative content analysis approach was undertaken whereby data were inductively analysed and summated to identify the key strategies used to achieve the delivery of exceptionally good general practice care, which were subsequently abstracted as a new level of the IDEAL framework.

**Results:**

In total, 222 individual factors contributing to exceptional care delivery were collated and abstracted into the framework. These included specific behaviours (e.g., patients providing useful feedback and personal history to the provider), structures (e.g., using technology effectively to support care delivery (e.g., electronic referrals & prescriptions)), processes (e.g., being proactive in managing patient flow and investigating consistently delayed wait times), and contextual factors (e.g., valuing and respecting contributions of every team member).

**Conclusion:**

The addition of concrete and contextual strategies to the IDEAL framework has enhanced its practicality and usefulness for supporting improvement in general practices. Now, a multi-level systems approach is needed to embed these strategies and create an environment where excellence is supported. The refined framework should be developed into a learning tool to support teams in general practice to measure, reflect and improve care within their practice.

**Supplementary Information:**

The online version contains supplementary material available at 10.1186/s12875-024-02352-1.

## Background

To-date, efforts to improve healthcare have been deficit-based; focused on learning from incidences of harm [1]. While many initiatives informed by this approach (referred to as Safety-I) have been developed, a lack of meaningful change [2, 3] in quality and safety of care is evident across healthcare settings including primary care [4, 5], a domain targeted less frequently than acute care [6, 7]. The heterogeneous ecology of primary care makes implementing improvement efforts challenging [8–11]; as such, solutions are needed that reflect the complex nature of the systems involved in providing care [12, 13]. In recent years, an emerging quality and safety paradigm has shifted our focus from deficit-based (“when things go wrong”) to asset-based improvement (“when things go right”; referred to as Safety-II) [1, 14]. Consistent with safety-II and other asset-based efforts (e.g., Learning from Excellence [15]) is an improvement approach referred to as positive deviance (PD) [15].

Having emerged in the field of international public health [16], the PD approach involves identifying, and subsequently, learning from individuals whose distinct behaviour(s) allows them to succeed where others in the community fail [16, 17]. Recently, the use of PD has emerged in healthcare as a means of learning from individuals, teams or organisations that perform exceptionally well compared to others, despite facing similar challenges [14, 16]. Central to the ethos of PD is the formulation of solutions from within the community [18], which are typically more acceptable and feasible within existing resources, and thus, are more likely sustainable and transferable elsewhere [16, 19].

In primary care, PD has been gaining momentum [20] and early applications have been promising, generating improvements in various healthcare-related outcomes [20–23]. A strength of this approach is its emphasis on uncovering both the concrete strategies used to perform exceptionally well, and the latent and abstract factors that support their delivery (e.g., organisational culture) [24]. By uncovering ‘what’ specific strategies positive deviants use to succeed as well as ‘how’ these are actually delivered [25], the PD approach intrinsically integrates important contextual information into understanding best practice [26]. Much of the difficulty of producing improvement lies in the enormous complexity of healthcare systems, including their challenging social, institutional and political contexts [27]. Acknowledging and attending to this cultural context is vital if improvement interventions are to succeed [28].

A recent review [20] synthesised applications of the PD approach in primary care to develop a framework of factors associated with positively deviant care outcomes; the Identifying and Disseminating the Exceptional to Achieve Learning (IDEAL) framework. More recently, this theoretical framework has undergone further qualitative refinement to examine its comprehensiveness, validity, and applicability in a novel context [25, 29]. The IDEAL framework, and other theories of high-performing primary care [30, 31], can be considered mid-range theory; theory that considers a specific phenomenon and involves a small number of concepts [32] delimited in their area of application, functioning at a level between ‘minor working hypotheses’ and ‘master conceptual schemes’ [27]. While high-level conceptual factors (e.g., patient activation) and mid-level subfactors (e.g., behaviour change) within the IDEAL framework [20] are useful for understanding a problem and developing interventions [27], identifying the specific downstream strategies, tools, processes and contextual factors provides more actionable guidance.

In addition, as core stages of the PD approach involve uncovering strategies that enable individuals to outperform others, and subsequently, sharing these strategies with others in the community [16], there is value in identifying, and disseminating, the specific lower-level strategies that are actually used in practice to achieve exceptional care delivery. Integrating practical guidance into the IDEAL framework will expand its usability, which is important as improvers, practitioners, and others at the sharp end, are interested in theory to the extent that it can help them improve their practice [27]. Accordingly, this study, comprising a secondary content analysis of semi-structured interviews with patients and practice staff, aims to extend our understanding of how exceptional care delivery is achieved in general practice [20]. Specifically, we sought to: identify and collate the specific strategies, behaviours, processes and tools used to support the delivery of exceptionally good patient care in general practice; and to abstract the identified strategies as an additional level of the IDEAL framework.

## Methods

This study is reported in accordance with the Consolidated Criteria for Reporting Qualitative Health Research (COREQ) [33]. Ethical approval for the original study was obtained from the University of Galway’s Research Ethics Committee (ref.2021.01.012).

### Study design

This study comprises a secondary analysis of previously conducted interviews with general practice stakeholders that focused on; identifying the factors and subfactors that support exceptional care delivery, and testing and refining the IDEAL framework [34]. While this original study produced a valuable framework for understanding exceptional care delivery, it was considerably conceptual or abstract in nature [27]. To enhance the IDEAL framework further, the current study sought to uncover the specific strategies that are actually used in practice to achieve these important aspects of care (i.e., the factors and subfactors), as integrating practical guidance into the framework provides more actionable intervention targets and highlights key mechanisms for change, thus, producing a holistic systems-focused framework for transforming primary care [27]. So in brief, while the previous study identified ‘what’ factors are important, this study identified ‘how’ these factors can be achieved. For the purpose of this study, any identified strategies, behaviours, organisational processes, tools, and contextual factors that target the previously identified factors were referred to collectively as ‘strategies’.

Accordingly, as the interview data were rich enough [35], a secondary analysis was undertaken to collate the practical strategies described by participants. A secondary analysis was suitable as this study’s research question, data collection and analytic techniques were sufficiently close to those of the primary research study [36]. Bradley et al. [26] have proposed a framework for applying PD principles in healthcare settings, which involves identifying positive deviants (Stage 1) and using qualitative methods to identify strategies that help them succeed (Stage 2), the efficacy of which are then tested statistically in larger, more representative samples (Stage 3), and finally, effective strategies are disseminated to others (Stage 4). This study aligned with Stage 2 [26], by employing qualitative inquiry to generate hypotheses about strategies that support positively deviant general practice. As noted previously [34] due to disruptions to Irish general practice caused by the COVID-19 pandemic [37] and a lack of publicly available performance data for general practice in Ireland [38], a modified approach informed by PD principles was taken. This involved investigating key general practice stakeholders’ perceptions and experiences of exceptionally good care delivery, as opposed to identifying exceptional performers and studying their performance.

### Theoretical underpinning

Safety-II principles also informed our approach, through a focus on everyday performance variability, consideration of the whole system, and emphasis on building adaptive capacities [39, 40]. Finally, Clinical Microsystems Theory (CMT), a systems approach to change that recognises the complexity of healthcare, inspired our methodology [41], and so, care was evaluated across multiple system-levels, including the clinical microsystem (i.e., practice team). The five levels of the general practice system explored, include: (1) the patient-level, the individual receiving care in general practice; (2) the provider-level, the individual directly providing patient care in general practice; (3) the microsystem (team)-level, the group of professionals working together to provide care to discrete populations of patients in general practice; (4) the mesosystem (practice)-level, the general practice, including its physical environment and how it is managed; and (5) the macrosystem-, network-, and national-level, the organisation of general practice services within the community and its interface with secondary care, as well as policies and support impacting general practice at a national level [41].

### Recruitment and participants

To ensure a heterogeneous sample in relation to age, gender, and profession/role [42], opportunistic and maximum variation purposive sampling were employed [34]. Participants included adults with experience in receiving (patients), providing, or managing general practice care in Ireland (General practitioners (GPs), practice nurses, practice managers). Serving as the first point of contact within the healthcare system, general practice provides approximately 29.1 million consultations annually [43] to public (i.e., patients with free access to general practice care as holders of either a General Medical Services (GMS) card or GP only card [43])) and private patients in Ireland. Primary care services are notably diverse, with practices varying considerably in terms of size, location, services, and team composition [44]. Recruitment materials were shared on social media and with relevant university departments, research networks and professional bodies in August 2021. To address participation barriers [45] and incentivise participation, all participants were entered into a prize draw for 8 gift vouchers. As shown in Table 1, a total of 33 participants were recruited and interviewed, with interviews lasting for an average of 44 min in duration (range, 15–90 min).

**Table 1:** Characteristics of participants

Characteristic	Patient *N* (%^a^)	GPs, nurses, managers *N* (%^b^)
***Gender***		
Female	9 (64.3)	15 (79)
***Age***		
20–29	3 (21.4)	1 (5.3)
30	3 (21.4)	5 (26.3)
40–49	3 (21.4)	6 (31.6)
50–59	3 (21.4)	3 (15.8)
> 60	2 (14.3)	4 (21.1)
***Practice experience (years)***		
< 5		2 (10.5)
5–10		4 (21.1)
11–15		6 (31.6)
16–20		4 (21.1)
> 20		3 (15.8)
***Average visits to the GP (per year***		
< 1	2 (14.3)	
1–2	8 (57.1)	
> 2	4 (28.6)	
***Primary care setting/location***		
Rural	7 (50)	4 (21.1)
Urban	7 (50)	14 (73.7)
Mixed		1 (5.3)
***Role in primary care***		
General Practitioner		13 (68.4)
Practice nurse		4 (21.1)
Practice manager		2 (10.5)

^a^Percentage of the patient sample (*N* = 14)

^b^Percentage of the GP, practice nurse and practice manager sample (*N* = 19)

### Data collection

Data were collected using semi-structured interviews. Questions and probes examined exceptional care at the five system levels (i.e., the patient, provider, team, practice, and macro, network and national environment). ‘Exceptional care’ was conceptualised as the delivery of an outstandingly high-quality of care that is perceived as effective, safe, efficient, patient-centred, timely, and equitable to an exceedingly high standard [34]. This meant that participants’ perceptions, understandings and previous experiences of care in general practice were elicited to identify factors and characteristics of an exceptional patient, provider, team, practice and external care environment (i.e., hypothetical positive deviants – those whose performance generated positively deviant care outcomes). In this sense, the study assumed that all participants had some understanding of exceptional care delivery through previous experiences of receiving, providing, managing, observing or discussing care with others, and would be able to reflect upon and identify aspects of these experiences that supported or characterised its delivery. The interview guide was piloted with a patient (Female; 6–8 visits to the GP a year; 30–39 age group; attends an urban practice) and GP (Female; 13 years working in general practice; 40–49 age group; works in an urban practice), and adapted as needed (see Additional file 1). ROM conducted the interviews by video call (Zoom©) at a time convenient for the participant from August 2021 until November 2021, when data saturation was achieved (i.e., no new categories shared by at least two participants in two consecutive interviews [46]) in both groups (patients and practice staff) [47]. Some participants were known to ROM (i.e. the first author: female PhD-level health services researcher), whose reflexivity may have been affected by her own experiences of receiving primary care and conducting related research in general practice. The interviewer had a relatively balanced ‘insider-outsider’ status with participants; having a shared understanding of primary care delivery, the interviewer was an ‘insider’ with practice staff, but was an ‘outsider’ to the direct provision of care in practice. Moreover, the interviewer shared an ‘insider’ status with patients in that their experience of primary care has predominantly been as a patient [48].

### Data analysis

**Fig. 1:**
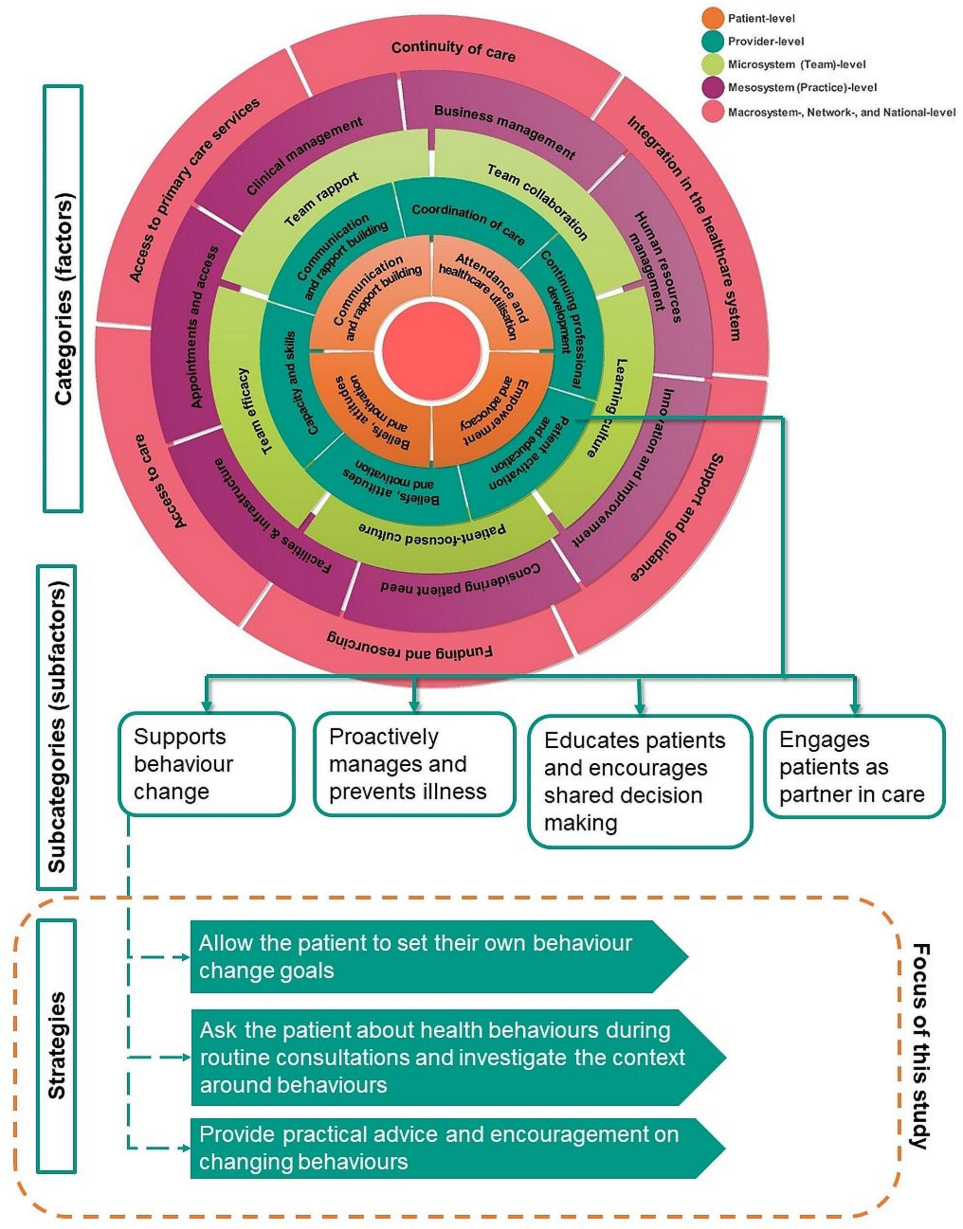
Example of categories, subcategories and strategies in the IDEAL framework

Interviews were digitally recorded, transcribed verbatim, and imported into NVivo 12 (QSR International Pty Ltd., 2018) [49]. In brief, as the previous study (see supplementary material in O’Malley et al. [34] for full procedure) sought to test and refine the original IDEAL framework [20] of factors associated with PD in general practice, a directed content analysis approach was adopted [50–52], as it allowed for the previously developed main categories of the IDEAL framework [20] to be deductively and inductively refined [52], and for newly emerging subcategories to be inductively analysed [34, 50]. During this analysis, several lower-level practical strategies that achieve the aforementioned categories were described, however, as the collation of these strategies was outside the aim of the previous study’s research question, they were not explored further. Collating and abstracting these strategies into the IDEAL framework compliments the previous study’s analysis and further enhances the framework’s capacity for informing improvement [27]. The goal was to deliver a framework of higher level factors (areas of general practice that support exceptional care delivery), mid-level subfactors (important elements of these factors), and now, lower-level strategies (practical strategies that target these important subfactors, and thus, factors). As seen in Fig. 1, this final lower-level is the focus of this study.

Accordingly, the current analysis adopted a summative content analysis (SCA) approach [52], as it allows for an interpretation of both the words and the frequencies in which stakeholders described the key strategies. A latent approach to the SCA was adopted, which examines the inherent meaning underlying the strategies, along with the context of their use and their tacit implications for practice [52–55]. While a directed content analysis approach was employed in the previous study to deductively test the previously defined theory [34], it was deemed unsuitable here as the current study’s aim focuses on identifying novel strategies inductively from the data. Of note, both analyses employed aspects of inductive reasoning, and elements of a SCA were used in the previous analysis to identify the frequency at which categories were reported.

Coding was performed in three steps. As part of the pilot coding, the first author coded data under 15% of the previously identified subcategories [34] inductively, which allowed for codes and strategies to be identified directly from our data [51, 56]. Pilot coding was reviewed as a team, and changes were made as necessary via consensus. Second, the first author coded data under the remaining 85% of subcategories, with a concurrent rechecking and revising of strategies [57]. Following the main analysis, similar or dissimilar strategies were combined appropriately [58] as far as was practical [51]. Important contextual information around how these success strategies are achieved was incorporated in two ways; by integrating contextual information into the strategies as they were coded (e.g., “adhere to the agreed treatment plan as best as they can” has the practical strategy of adhering well to one’s care plan but acknowledges that patients have different levels of capacity), and by coding contextual facilitators as strategies (e.g., ensure that every team member’s role and responsibilities are clarified, treat each other with dignity and respect), which enable the use of more direct strategies (e.g., coordinate effectively to provide seamless services). Codes and strategies were refined throughout the analysis, and coding decisions made by the first author were discussed regularly with the team until consensus was achieved [59]. Strategies were presented in tables with exemplar quotations from participants. Frequencies, commonly used in SCAs [52], were collated to highlight the frequency at which participants described each strategy [60]. Similar to previous research that explored factors and strategies associated with high performance in healthcare [61], an interactive figure was made in PowerPoint to present the factors, subfactors and strategies of the revised IDEAL framework.

To ensure trustworthiness, procedures by Lincoln and Guba were followed [62]. For example, a reflective journal and audit trail were maintained to support the study’s confirmability and dependability [63, 64], and transcripts were read several times and strategy choices were discussed until reaching consensus [65]. Further, member checking of the initial transcripts enhanced credibility, and transferability was ensured by integrating several perspectives and developing detailed research descriptions [63].

## Results

### Refinements to framework

Overall, 1,926 meaning units, or individual factors supporting exceptional care delivery, were coded. The current study abstracted 222 newly coded strategies that help achieve exceptional care delivery into the framework, and the original IDEAL framework was refined into 28 categories, 103 subcategories (from 28 categories and 104 subcategories [34]), and now, 222 newly coded strategies. An interactive PowerPoint figure presenting the factors, sub-factors and strategies of the revised IDEAL framework is available in Additional file2.

### Strategies identified

A comprehensive list of categories, subcategories and strategies emerging from our analyses with supporting quotations are presented in Additional file 3. The following sections report the most commonly described subcategories of each category with some of the identified strategies. Figure 1 presents an example of the three levels of the refined framework.

#### Patient-level

The patient’s sense of **empowerment and their ability to self-advocate** (91%) for their healthcare needs and preferences, comprised four subcategories (see Table 2 for sample strategies). Strategies in the ‘feeling empowered as a patient’ (64%) subcategory, for example, focused on *demonstrating agency and ownership over healthcare decisions* and *clearly indicating one’s own needs and preferences*.

**Table 2:** An example of categories, subcategories and strategies coded at each system-level

Category (%^a^)	Subcategory (%) Exemplar quotation^b^	Strategies
**Patient-level**		
**Empowerment and advocacy (91%)**	Feels empowered as a patient (64%) *“…they become partners in whatever is going on. So you see them as a partner like an equal. (GP6)”*	○ Demonstrate agency and ownership over own healthcare (e.g., following up on results, bringing advocate to consultation).
		○ Clearly indicate their healthcare needs, preferences and expectations to the provider.
		○ Willing to take part in practice research and open to having medical students present.
	Engages in shared decision-making and care planning (42%) *“…willingness to undertake shared decision making and shared care… the risks and benefits of various treatments. (GP1)”*	○ Adhere to the agreed treatment plan as best as they can and try to make suggested lifestyle changes.
		○ Are willing to build a shared understanding with the provider and engage in shared decision making.
	Recognises important health issues and provides accurate and useful information (36%) *“…they’d be clear in their symptom description, and they’d be clear in how their symptoms are affecting them… (GP5)”*	○ Provide clear and useful feedback and history to their provider and actively engage in care planning.
		○ Can differentiate between urgent and non-urgent issues (e.g., identifying non-serious headaches as distinct from those requiring further investigation).
	Actively seeks health information (27%) *“…ask the doctor any concerns, any questions you have about the treatment. (P5)”*	○ Actively engage during the consultation and ask for clarification and expansion as necessary to facilitate understanding of health condition and treatment plan.
		○ Independently research relevant health information and seek the provider’s input as necessary.
**Provider-level**		
**Communication and rapport building with patient (100%)**	Listens to the patient (73%) *“…they’re listening attentively to what you’re saying…and they are giving it the credence that it deserves. (P13)”*	○ Give the patient their full attention and make the patient feel like they are listening and not rushing through the consultation.
		○ Listen attentively to what the patient is saying and give it credence.
	Reassures the patient and alleviates their concerns (61%) *“…ask them what they think is going wrong or going on, what are they most worried about, and then addressing those factors as well. (P9)”*	○ Try to make patients feel comfortable and use techniques to put them at ease (e.g., making small talk), especially during difficult procedures.
		○ Identify the patients’ concerns and reassure them where they can (e.g., explain patients concerns definitively).
	Builds a supportive relationship and gets to know the patient (61%) *“…they would know your history, they’d know your family’s history. (P5)”*	○ Get to know the patient on a personal level and find commonalities.
		○ Build a long-term relationship with the patient and leverage it to provide necessary care.
	Trusts the patient and treats them with dignity and kindness (52%) *“They’re nice, friendly…they make me feel comfortable to talk about everything. (P6)”*	○ Treat the patient in a respectful, friendly and caring manner.
		○ Communicate in an open and honest way with the patient.
		○ Trust the patient to follow action plan as best as they can.
	Communicates effectively with the patient (42%) *“…finding a level that’s appropriate for the patient… and knowing how to pitch it. (GP7)”*	○ Demonstrate clear and direct communication and focused body language.
		○ Pick up on patient cues and tailors their communication style to suit the patient.
**Clinical Microsystem (Team)-level**		
**Team collaboration (97%)**	Collaborates to provide integrated care (70%) *“…our roles are different but they don’t work in isolation in parallel, they are integrated all the time. (GP11)”*	○ Coordinate effectively to provide seamless services to patients (e.g., allow admin staff to pass on messages to patients, engage in results coming back as a team).
		○ Refer patients within the team and help out colleagues to provide integrated care to patients (e.g., asking questions).
	Uses structured lines of communication (67%) *“…direct means of communication between, the doctors, the consultation rooms, reception… important for it to be discreet as well. (P4)”*	○ Maintain open methods to communicate, debrief and share patient information regularly amongst the team (e.g., whiteboards).
		○ Ensure communication about patients amongst the team remains respectful and discreet.
		○ Use technology to support communication and teamworking (e.g., daily electronic tasks, Whatsapp).
	Has regular practice meetings (55%) *“…weekly meetings, monthly meetings, they have to be listened to, you have to feedback, and vice versa. (GP4)”*	○ Operate regular practice meetings to ensure good management of the practice, with more regular meetings as issues arise.
		○ Ensure engagement with meetings and communication of discussions from meetings to all staff (e.g., full team or parts of the team are present, sharing meeting minutes).
**Mesosystem (Practice)-level**		
**Facilities and infrastructure (100%)**	Provides a pleasant and safe environment for staff and patients (91%) *“…it’s modern, it’s quite a nice building, very handy location… and it’s very spacious, airy, feels very clean, no carpets, everything, surfaces clean, clean, clean. (P7)”*	○ Maintain a well-organised and up-to-date bright, spacious and aesthetically pleasing environment (e.g., plants, art) that is conducive to patient flow and allows for flexibility in organisation.
		○ Try to create a comfortable environment for patients (e.g., water, comfortable seating, and TV or toys for kids) with adequate bathroom and baby changing facilities and potential for a separate waiting space (e.g., for patients with sensory issues).
		○ Create an environment suited to staff needs, including a comfortable work space, staff room and cafeteria, and staff bathroom facilities.
	Uses IT systems effectively to support the delivery of care (79%) *“We do use technology a lot to inform patients and make announcements and try and manage our workload… (GP2)”*	○ Make effective use of different functionalities of the system to optimise care and provide feedback on the tool as necessary (e.g., recall, safety pop ups, audit, tracking and accounting features).
		○ Provide a practice website which allows for online booking of appointments, ordering of prescriptions and payment.
		○ Use technology to communicate with patients and further care services (e.g., text service, electronic prescriptions to pharmacy, electronic referral to physiotherapists).
	Provides parking facilities and good accessibility (52%) *“Car parking is a big thing, wheelchair access, a lift, accessibility. (GP4)”*	○ Provide good, safe accessibility in the practice, with a wheelchair ramp, lift and other methods of ensuring good accessibility for different needs.
		○ Provide clearly marked parking facilities for patients and staff.
	Has necessary equipment and resources (39%) *“…the right equipment and technology to be able to support a good practice. (GP3)”*	○ Ensure all necessary equipment is available and easily accessible within the consultation room or practice.
		○ Provide comprehensive diagnostic facilities at the practice for bloods, ECHO and other services.
**Macrosystem-, Network- and National-level**		
**Integration in the healthcare system (Network) (91%)**	Lines of communication are established and information is continuous (76%) *“…good channels of communication setup between primary care doctors and our secondary care colleagues, where we can ask questions or ask for opinions. (GP4)”*	○ Develop and use direct and open lines of communication to secondary colleagues (e.g., to ask questions about referrals, get estimated timeframe).
		○ Send appropriate and accurate information back to primary care promptly, with an appropriate amount of detail and plan for care.
		○ Develop patient health records that can be accessed by all providers and share relevant information promptly with secondary care.
	Access to specialists and diagnostics is timely (39%) *“…prompt access…whether it’s investigations or specialist consultations. (GP5)”*	○ Identify and maintain referral pathways with prompt access directly to secondary care specialists.
		○ Identify and maintain direct access scopes and referral pathways with prompt access to diagnostics.
	Primary care is core of an integrated system (27%) *“…properly interconnected with these other services…it’s just one kind of seamless route. (P10)”*	○ Integrate PC better in the infrastructure to provide seamless links between services (e.g., integrating primary care into hospital care services).
		○ Use primary care resources as central to healthcare, in line with Government policy.
	Providers build relationships and have regular interfaces (21%) *“…there has already established relationships between primary and secondary care, and it is nurtured. (GP4)”*	○ Take part in common meetings and Continuing Medical Education schemes where providers can work together and develop mutual understanding of different care settings.
		○ Practice staff build rapport and have good working relationships with secondary care providers and services.

^a^Percentage of patients who reported a strategy at this category

^b^For the participant identifiers, ‘P’ denotes a patient, ‘GP’ denotes a general practitioner, ‘PN’ denotes a practice nurse, and ‘PM’ denotes a practice manager

**Communication and rapport building with provider** (79%), the patient’s role in communicating and building an interpersonal relationship with their provider, also had four subcategories. To support ‘building rapport and a supportive relationship’ (52%), for example, patients might *treat their provider and practice staff with respect and pleasantness* and *try to develop a good relationship with their provider*.

The patient’s **beliefs, attitudes and motivation** (76%) around health and healthcare, and expectations around seeking care, had four subcategories. Strategies that enable ‘having reasonable expectations around healthcare (30%)’ include *being understanding of staff and if appointments are late* and *making good use of time in the appointment and practice*.

Finally, the patient’s **attendance and healthcare utilisation** (58%) of primary care services, comprised two subcategories. One of these, ‘Turning up on time’ (42%) was achieved by *turning up on time to scheduled appointments or informing the practice if they will be late/need to reschedule*.

#### Provider-level

**Communication and rapport building** (100%), the provider’s role in communicating and building an interpersonal relationship with their patients, comprised five subcategories. Strategies that support the subcategory, ‘listening to the patient’ (70%) involved *giving the patient their full attention* and *listening attentively to what patient is saying*.

**Coordination of care** (91%), efforts to coordinate patient care activities and share information among healthcare professionals, had four subcategories. Strategies in the ‘coordinating good future care’ (82%) subcategory involved *understanding and making effective use of the referral infrastructure* and *clarifying different options for care and the referral process*.

The **beliefs, attitudes and motivation** (85%) of the provider around healthcare and its delivery, comprised four subcategories. Strategies involved in ‘taking a holistic approach to healthcare’ (49%) included *treating the ‘whole person’ and considering the patient’s context, history and background* and *looking past presenting symptoms to uncover the cause.*

The **capacity and skills** (82%) of the provider, what they are capable of doing within their knowledge, skills and workload, had five subcategories. ‘Managing time effectively and being organised’ (58%), for example, involved *actively managing time within the consultation while allowing for patient’s concerns to be safely discussed* and *organising time and tasks to be performed for the day*.

**Patient activation and education** (73%), efforts to activate patients to enhance their understanding and willingness to be a partner in care, comprised four subcategories. Strategies that support providers in ‘educating and encouraging shared decision-making’ (33%) involved *identifying and considering what the patient wants and encouraging shared decision-making* and *providing unbiased reputable information on their condition and treatment options*.

**Continuing professional development** (33%), the provider’s propensity to engage in learning activities to develop their understanding, knowledge, and skills, comprised three subcategories. ‘Self-reflecting and wanting to improve’ (21%) requires that providers *are interested in learning new things and developing specialisations* and *reflect on their practice and look at how to get better*.

#### Microsystem (Team)-level

**Team collaboration** (97%), the team’s propensity to engage in programmed opportunities for teamwork and to work cooperatively, comprised three subcategories. The subcategory ‘Collaborating to provide integrated care’ (70%) was achieved by *coordinating roles effectively to provide seamless services* and *referring within the team and helping out colleagues to provide integrated care.*

The shared beliefs of team members around the **team’s efficacy** (94%) and ability to perform tasks effectively, comprised four subcategories. Strategies that support ‘valuing and trusting team members’ (91%) included *valuing and respecting the contributions of every team member* and *having a teamwork orientation and trusting colleagues to perform tasks effectively.*

The quality of **team rapport** (88%), and how it shapes team interactions, culture, and collaboration, comprised four subcategories. To ‘create a supportive and friendly working atmosphere’ (64%), for example, teams might *offer each other emotional support and support those who are struggling* and *foster a happy supportive working environment where everyone is treated with kindness and friendliness*.

**Patient-focused culture** (55%), the extent to which the team’s culture focuses on patient values, preferences and needs, comprised two subcategories. Strategies that ‘make the patient feel welcome and valued’ (39%), for example, involved *treating patients with friendliness and responding in a manner suited to the patient* and *making the patient feel important, involved and valued as part of their team.*

**Learning culture** (42%), the extent to which the team’s culture emphasises continuous learning and improvement, had three subcategories. One subcategory, ‘learning from when things go wrong’ (27%), involved *learning from incidents as a team to mitigate future risk* and *fostering a no blame culture where people feel comfortable reporting errors.*

#### Mesosystem (Practice)-level

The **facilities, infrastructure** (100%), and available resources of the GP practice, comprised four subcategories. To ‘provide a pleasant and safe environment’ (91%), for example, practices should *maintain a well-organised and up-to-date bright, spacious and aesthetically pleasing environment* and *create a comfortable environment for patients with adequate bathroom, baby changing and other facilities.*

**Appointments and access** (91%), systems at the practice that enable the efficient allocation of appointments and access to care, comprised five subcategories. Strategies that ‘actively facilitate access to care’ in the practice (58%), for example, involved *sending reminders of appointments* and *making it clear to patients how to check in to the practice*.

**Clinical management** (85%), the management of clinical systems and processes within the practice, and use of standardised care protocols, comprised three subcategories. One of these subcategories, ‘utilising robust systems for clinical management’ (70%), involved *using technology effectively to support care systems* and *maintaining efficient and prompt systems for communicating with patients.*

The category **considering patient need** (73%), efforts by the practice to address the needs and preferences of their patients, included four subcategories. Strategies that ‘Safeguard patients’ privacy’ (39%), for example, involved *creating a private area to communicate with reception and ensuring communication is discreet* and *maintaining the patient’s right to privacy while in the practice (e.g., playing music in hallways).*

**Business management** (67%), the extent to which the practice is managed like a business, with the effective management of workload and high levels of organisation, comprised four subcategories. Strategies in the ‘maximising scheduling and proactively managing wait times’ (48%) subcategory involved *maximising scheduling and allocating an appropriate number of appointments each day* and *being proactive in managing patient flow and wait times*.

**Human resources management** (61%), the governance of staff within the practice, including the management, training and appreciation of staff, comprised three subcategories. ‘Effectively managing staff’ (52%), for example, involved *delegating responsibilities, tasks and leadership roles to appropriate staff members* and *clearly and appropriately scheduling staff and ensuring staff contracts are maintained.*

The organisation’s emphasis on pursuing **innovation and improvement** (52%) and deliberately implementing changes, comprised four subcategories. ‘Eliciting staff and patient feedback’ (33%), for example, involved *using proactive methods to elicit patient and staff suggestions or complaints* and *trying to implement patient and staff input and explaining if solutions are not possible*.

#### Macrosystem-, network- and national-level

The ease at which patients can **access necessary specialty primary care services** (79%) comprised two subcategories. Strategies in the ‘Primary services are located in close proximity’ (52%) subcategory included *developing primary care centres where services are located within the same building* and *ensuring the practice is located near or has good links with other primary care services*.

The integration of primary care within the community and the **continuity of care** (64%) received by patients, comprised four subcategories. ‘Lines of communication are established and information is continuous’ (36%) in the primary care environment was achieved by *maintaining accessible lines of two-way communication with other primary care providers* and *keeping patients’ records updated with new information and sharing relevant information with other providers*.

**Integration of primary care in the healthcare system** (94%) and the ease at which patients can access services outside of primary care, comprised four subcategories. Ensuring ‘lines of communication are established and information is continuous (76%)’ with secondary care, involved *developing and using direct and open lines of communication to secondary colleagues* and *sending appropriate and accurate information promptly back to primary care*.

**Access to care at a national level** (73%), the accessibility of primary care services for all patients within a given country, comprised three subcategories. Strategies that support ‘timely access to care for all’ (42%) included providing *timely access to care (including public patients)* and *free universal healthcare to all people within the country*.

**Funding and resourcing** (67%) from the health system and national agencies comprised four subcategories. ‘Managing GP workforce and recruitment’ (48%), for example, was achieved by *training and maintaining a sufficient number of the right staff* and *creating a good working environment and work-life balance for general practice staff.*

Tangible **support and guidance** (49%) provided by the health system to facilitate the delivery of primary care, comprised four subcategories. ‘Providing training and support for general practice (30%)’, for example, involved providing *standardised education for all healthcare roles in general practice* and providing *practical guidance to staff and developing national repositories of information*.

## Discussion

Achieving truly exceptional care requires the realization and implementation of key strategies that make it possible. This study comprised a secondary analysis of previously collected interview data that explored the key factors and subfactors that characterise exceptional care delivery. In total, 222 new strategies that are used to achieve the previously identified factors of exceptional care delivery were identified and abstracted into the IDEAL framework [34]. The addition of these strategies enhances the IDEAL framework’s practicality and usefulness for informing the development and implementation of future improvement efforts, and ultimately, for achieving truly exceptional care delivery in general practice.

### Understanding the strategies that facilitate exceptional care delivery

A variety of strategies, behaviours and contextual factors were identified at the patient- and provider-levels, many of which show promise for improving care. Several of the identified strategies have been associated with exceptional primary care in the wider quality research literature, including, for example, strategies that help build a trusting partnership (e.g., rapport [66], respect and listening [67]) and develop tailored care plans (e.g., goal setting [66], discussing health behaviours [68]). Moreover, as many strategies were actively being used by participants, it is possible that they are feasible to implement within the current general practice landscape [16, 25]. Further, as these strategies were generated by patients and providers on the front line across different settings (i.e., a mix of rural and urban practices across Ireland), there is a greater likelihood that they will be acceptable to others in the community, which in turn makes it more likely that they are adopted and sustained [16, 26]. While links with existing research suggest that the identified strategies may be useful, feasible, and acceptable to patients and providers in general practice, examining these strategies statistically in larger samples would contribute further evidence of their efficacy in achieving exceptional care delivery, which is valuable information for improving uptake amongst patients and providers [26].

However, while these strategies may show promise for improving care, careful consideration must be given to ensure their effective and equitable uptake among patents and practice staff. For example, patient-level strategies may be more attainable to patients with greater resources given that socioeconomic status predicts a patient’s sense of empowerment [69]. Further, as identified by this study, a core strategy of an exceptional provider relates to their ability to engage socially, psychologically or medically complex or vulnerable patients. This is particularly important for primary care providers, who are uniquely positioned to address social determinants of health through a focus on recognising and meeting patients’ needs in the community [70]. While the identified patient-level strategies provide actionable guidance for motivated patients looking to build their capacity for engaging in healthcare, it must be recognised that many strategies will not be attainable for the most vulnerable of patients. In this sense, it is a core responsibility of the provider to proactively work with vulnerable patients to help them reach a capacity where they can engage with these strategies (e.g., take part in shared-decision making, make lifestyle changes). Moreover, as there is evidence to suggest that many of the provider-level strategies may help engage and meet the needs of particularly vulnerable patients (e.g., establishing trust and showing respect, getting to know the patient and building relationships, being non-judgemental and understanding the role of social determinants of health [71, 72]), an examination of which strategies are most effective, and acceptable to patients, in engaging vulnerable patients will help guide providers looking to support and empower those who need it most.

Accordingly, if we know that partnering with patients can increases their sense of empowerment [73], providers should view the ‘exceptional patient’ as the desired outcome of their caregiving, particularly for the most vulnerable of patients [74]. Interestingly, many strategies identified were bidirectional, performed and reinforced by both sides of the patient-provider dyad (e.g., trust, kindness, listening), elucidating the reciprocal partnership formed between the provider and patient [75]. The reciprocal nature of this partnership [76], suggests that targeting strategies that build the provider’s capacity to engage patients may empower both sides of the therapeutic dyad [77]. Previous efforts have successfully transferred patient-centred skills to providers [78], positively impacting patient behaviour [79] and health outcomes [80]. So, if providers or health systems can help patients become ‘exceptional’ [81], harnessing the provider may empower both the provider and patient [82]. Nonetheless, support from higher system-levels would help create the environment where excellence is supported [83] and provide primary care providers with the necessary tools to transform practice [84]. Future research should explore methods for facilitating a positive cycle of learning between the patient and provider, whereby providers are supported to both achieve the identified provider-level strategies and support patients to achieve strategies that empower them.

At the team-level, while many strategies represented tangible behaviours that teams perform to deliver exceptionally good care (e.g., operating regular meetings), most strategies comprised cultural or relational behaviours and beliefs that support the delivery of these strategies (e.g., valuing and respecting everyone’s contributions) [25]. For example, identified strategies were consistent with relational team characteristics that facilitate practice improvement and high-quality healthcare (e.g., trust, respect, communication) [85]. Team relationships are key to positively deviant primary care [25, 86], and in complex adaptive systems like primary care practices [85], relationships form key levers for improvement [85]. The nature and extent of the impact of social relationships is generally referred to as ‘social capital‘ [87, 88] – a concept used by social scientists to highlight the pivotal nature of relationships [89]. Current strategies for improving quality in healthcare settings often emphasise individual role development and job descriptions [90, 91]. However, while specialisation is important, high-quality interactions and relationships are also needed to ensure that primary care practices can fully utilise the specialised skills of all its members [89]. Similarly, team beliefs were frequently addressed within strategies at this level, which is consistent with previous applications of PD in primary care (e.g., beliefs around team effectiveness, the patient [86] and learning [25, 86]). Interestingly, there is evidence to suggest that a team’s beliefs and their collaborative practice are reciprocally related [92], indicating a reciprocal positive gain spiral of beliefs and collaboration, the contextual and the concrete [82]. As proposed in the broader PD literature [24], in addition to targeting *what* teams do, harnessing cultural and social behaviours is a valuable strategy [25] to improve *how* they do it. These beliefs, values, behaviours, and interactions culminate in the team’s ‘culture’ [93], which, given its links with quality [94] is a promising target for enhancing an organisation’s effectiveness [95, 96]. While cultural strategies are typically more abstract and challenging to target, culture can be effectively improved [97] and many strategies bolstering culture are relatively feasible (e.g., having a coffee together [98]). Practice leaders need to appreciate that excellence is influenced by relating, and should dedicate time and space for building relationships and learning [85]. Future improvement efforts should harness the power of the team to diffuse innovations in primary care, and need to ensure contextual and cultural factors are being targeted alongside the implementation of more tangible, concrete strategies.

Similar to previous research, most practice-level strategies related to resources, organisational structure, and clinical and operational processes within the practice [67], referred to as the practice’s core [99]. An effective core is critical to successful practice development [99], and many of these strategies help build capacity, such as those targeting administrative resources, access, staffing levels and mix [94]. As expected, a considerable number of practice-level strategies require additional resourcing, however, participants also described many strategies that could be implemented within existing resources (e.g., improving access signage). In addition to the practice core, a practice’s internal capability also comprises an adaptive reserve; features in primary care practice that enhance resilience [99]. While a robust core helps meet ordinary variations in care and maintain consistent care delivery, an effective adaptive reserve facilitates adaptation during times of change [99]. Importantly, the strategies identified herein help build a practice’s core, but also, bolster its adaptive reserve (e.g., developing learning cultures, supporting innovation [99]). Moreover, key to a practice’s adaptive reserve is the clinical microsystem, or practice team [100], and emerging strategies target practice-level characteristics needed to support high-performing clinical microsystems (e.g., establishing practice goals and expectations) [41]. The quality and impact of relationships amongst team members, referred to as social capital, is key to an effective adaptive reserve and clinical microsystem [99]. Functioning as CAS, primary care practices need social capital to succeed [89], as relationships are as important to the system’s success as the qualifications and capacities of the individuals themselves [89, 101]. The quality of care in primary care practices is a function of the quality of social capital among practice members [89], whereby, higher levels of social capital has been associated with better patient perceptions of quality [102]. Improvement in primary care typically identifies the physician as the locus for practice improvement [89], however bolstering the team’s social capital and capacity to function as an effective clinical microsystem is more likely to build adaptive reserve at a practice level. So, while organisational resourcing is needed to implement strategies that build the practice core [98], strengthening the practice’s internal capacity also requires the implementation of strategies that bolster the practice’s adaptive reserve. Given that practice-level strategies typically require additional resourcing, research is needed to determine what strategies at the practice-level are critical for performance, with consideration of both effectiveness and feasibility, which should subsequently be considered in the development or redesign of primary care practices.

Within the broader care environment, many strategies have been previously linked to more effective and efficient external care services, including, for example, relocating specialist services to the community, working as an integrated team with other providers, and improving communication lines to increase the availability of specialist advice [103]. In addition, research suggests that strategies at the national-level would bolster a high-performing primary care system, such as having fair and effective funding and administrative models [104, 105], maintaining a skilled general practice workforce [104–106] and supporting quality, learning, and governance at a national-level [104–106]. Governments want to do more with existing resources [105], and while many national-level strategies require considerable resourcing, cost-effective strategies were also uncovered. For example, including general practice in policy-making [107] and having educational interfaces for primary and secondary care providers [108] are feasible, yet effective strategies. However, it is important to note, that national-level strategies may reflect nuances of the Irish health system, where the involvement of primary care providers in policy making and the delivery of joint primary-secondary educational interfaces occur somewhat inconsistently. Yet, many prominent issues in Irish healthcare are similar to those experienced by other health systems [109]; for example, while joint events for general practitioners and specialist doctors are widely valued, they are infrequently delivered across many European countries [110]. Further, the fact that many of the identified national strategies are still recommended across primary care [109] indicates that they are still priorities in similar health systems. Policymakers need to ensure that they are bolstering strategies that provide an environment for exceptional care delivery to become commonplace at a national level, but given that health systems are highly context-specific, nuances of their individual health system need to be meaningfully considered in the selection, adaptation and implementation of strategies that bolster the whole health system [105].

In addition, some of the identified strategies may circumvent prominent health system issues, such as increasing the specialized nursing workforce [111] to reduce workload and workforce shortages [112]. Implementing national-level strategies can also establish and cascade systemic change across all levels of the health system [106]. For example, increasing the general practice workforce increases staffing locally, which allows for longer appointments, more time to engage patients, and thus, better patient outcomes [113]. Accordingly, a more integrated response that appreciates the inter-dependence of each part of the health system is needed [105]. As complex adaptive systems, primary care practices operate across multiple interconnected levels, and so efforts targeted at the provider- or practice-level only will not transform the whole system, and so far, have yielded only modest effects [106]. So while efforts targeting downstream strategies are a necessity for transforming the health system, they will fail to be actualized unless funders align their support with system-wide efforts [106]. In addition to implementing strategies that make excellence possible at a national level, policymakers should use existing educational and governance structures, such as Continuing Medical Education programmes [110], to support and encourage the implementation of provider-, team- and practice-level strategies that enable exceptional care delivery at a local level.

### Developing the IDEAL framework

This is the third study detailing the development and refinement of the IDEAL framework. Importantly, our process is embedded in practice and iterative [114, 115], moving from deductive to inductive, from theory to practice, which is core to good theory-building and contributes to the validity of factors proposed by this framework [114]. As well as strengthening the theory, the addition of newly identified strategies has enhanced the framework’s usability and practicality [27]. Mid-level theory, like the original IDEAL framework [34] is useful for understanding a problem and for planning and conceptualizing purposes [27], but is difficult to operationalize, and so, practical strategies are needed [116]. The strategies identified herein, which may be considered small theory, are purposefully practical and accessible [27]. The addition of concrete working models like these strategies is valuable for two main reasons: first, they specify the parts of an improvement programme intended to improve the phenomena under study, as well as the intervention’s expected outcomes and potential evaluation methods; and second, they suggest a ‘theory of change’, the assumptions about mechanisms that unite a programme’s processes and inputs to outcomes, along with the context needed for effectiveness. Thus, the newly refined IDEAL framework delivers a comprehensive and holistic approach to improving care delivery, that provides key intervention components (strategies) together with a narrative about the structures, behaviours, processes and contextual features (factors and subfactors) needed to achieve the aims of the intervention [27]. Now, additional research examining the framework is warranted to specify the specific factors and strategies that constitute key contextual facilitators of success and to uncover the specific mechanisms of action by which the factors and strategies influence exceptional care delivery [117]. Following this, additional research applying the IDEAL framework in novel contexts is needed to strengthen its validity and generalisability further, and to determine if strategies work in isolation or whether certain strategies, or combinations of strategies, are more or less effective for different patient groups (e.g., chronic versus acute illnesses), settings (e.g., rural versus urban, large versus small practices), and care processes (e.g., improving vaccine uptake versus managing long terms needs) [118].

### Strengths and limitations

The initial interview study [34] had a number of methodological considerations that are relevant here. One strength, for example, is having a broad sample in terms of location, age, healthcare role, and gender was important, as perceptions can differ based on these factors [119, 120] and getting the perspectives of practice nurses and managers along with GPs increased the applicability of findings [121]. However, it must be noted that while the sample also comprised patients from varying levels of socioeconomic status, it was not diverse in terms of ethnicity, which hinders the transferability of findings across patient populations [122]. Further, estimates suggest that patients in Ireland attend their GP approximately 4 times a year [43], which is higher than most patients in our sample. This may be partially explained by the fact that patients who regularly attend their GP tend to be older in age [123] and our sample had a relatively even distribution of age groups, with a greater representation of younger patients than the broader patient population regularly attending GP care in Ireland. Research applying the IDEAL framework in different patient samples is now needed to ensure the unique thoughts, values and perspectives of all patient backgrounds are considered in understanding and improving care delivery in primary care [122]. As noted previously [34], a completely true PD approach could not be completed due to COVID-related changes to practice [124] and data accessibility issues [38, 125]. However, we believe that our modified improvement approach has merit in identifying key success factors. Further, interview data were collected during the COVID-19 pandemic, which likely impacted participants’ interviewing experience [126], so efforts were made to build rapport with participants before interviews.

Further, secondary analyses can be limited given that original data were collected for other purposes [35]. However, having a good fit between the primary dataset and the research question explored here lessens this concern [127]. In addition, as the first author was personally involved in data production, they understood the relevant context needed to interpret data [36]. Nonetheless, the first author’s familiarity with the context surrounding the interviews could have led to oversight, and findings may have differed if the focus of interviews had been exclusively on identifying strategies [128]. Moreover, some strategies were noted by one or two participants only, which raises questions about generalisability. However, these strategies were discussed as a team and retained if deemed sufficiently distinct and useful. Further, although an examination into the strategies described by patients compared with practice staff would be valuable, differences in the perceptions of patients and practice staff was explored previously in the original interview study by the authors [34]. The resulting framework could be criticized for its length, which lead to a relatively brief and surface-level presentation of findings in this study. However, we sought to provide a robust toolkit of strategies to support improvement in general practice recognizing the benefits of having options or varied strategies available to support a common aim. Although many strategies have been suggested in separate research studies, they have not been combined as a holistic strategy to support learning in general practice [129]. Now, a feasible method of disseminating these strategies is needed.

### Implications for research, practice and policy

First, future research is warranted to explore the feasibility, usefulness and effectiveness of these strategies in larger representative samples [20, 26]. In line with the PD framework [26], newly identified strategies need to be disseminated to others in practice. One approach would involve developing a team-based learning tool, similar to the Manchester Patient Safety Framework [130], that would allow teams to both measure their capacity for delivering exceptional care and, subsequently, promote discussion around strategies for achieving improvement [131]. As discussed above, targeting the tool at practice teams would be an effective approach to enable change [132], as clinical microsystems support the effective uptake of innovation in primary care delivery [133].

In practice, organizational cultures that support teamwork and quality improvement contribute to achieving high quality care [134]. Practice leadership should create environments that support collective learning processes and practices [132], for example, by providing tools and processes that structure, facilitate or trigger teamwork [97]. Secondly, practice leadership should target the strategies identified here, many of which also enable the success of practice-based improvement initiatives [9, 135]. At a practice-level, using a tool such as that described above would support improvement, following adaptation for local context by the team [106].

At a policy-level, a new improvement approach is needed, that recognises the complex adaptive nature of health systems [106] and emphasises national-level policy reforms alongside downstream efforts. This includes the implementation of national-level strategies identified herein (e.g., investing in developing technological infrastructure, developing incentives that support high-quality care). First, a political commitment is needed to tackle the social, political, economic, and organisational structures that shape health systems, and optimize coordination of care at a network- and community-level [106], followed by support for local-level interventions that directly target staff or practice performance [106]. Governments need to support practice teams in achieving the strategies collated herein while also pulling the levers that make excellence possible at the national level.

## Conclusion

The strategies identified herein offer a promising strengths-based approach to improving care delivery in general practice. Primary care is at the core of an intrinsically interconnected system, and until all systems commit to embedding these strategies, improvement efforts will fail to realize their potential. If exceptional care is to become everyday care, we must create an environment where patients, providers and teams can build trusting and therapeutic relationships, learn and innovate together, and coordinate effectively to provide holistic family-centred care to people of every need.

### Electronic supplementary material

Below is the link to the electronic supplementary material.

Supplementary Material 1

Supplementary Material 2

Supplementary Material 3

## Data Availability

Availability of data and materials: All data generated or analysed during this study are included in this published article [and its supplementary information files].

## Abbreviations

COREQ: Consolidated Criteria for Reporting Qualitative Health Research
CMT: Clinical Microsystems Theory
GP: General Practitioner
IDEAL: Identifying and Disseminating the Exceptional to Achieve Learning
PD: Positive Deviance
PM: Practice Manager
PN: Practice Nurse
SCA: Summative Content Analysis
