# Supplementary material for: Strategies that facilitate the delivery of exceptionally good patient care in general practice: a qualitative study with patients and primary care professionals
Source: BMC Prim Care. 2024 Apr 27;25:141. doi: 10.1186/s12875-024-02352-1 (PMC11055247; doi:10.1186/s12875-024-02352-1)
Supplement: Supplementary file 1 — Supplementary Material 1 [file 12875_2024_2352_MOESM1_ESM.docx]

**Additional file 1.** Interview guide used during data collection

As you know, this interview will focus on exceptional care delivery in general practice, and when we talk about exceptional care, we refer to care that is outstanding compared to typically (good) care that is usually provided in general practice.

- The delivery of patient care is a complex process involving many different levels of the healthcare system including: the patient; the individual provider; the practice team; the practice management; to primary care services in the community; the extended healthcare environment; as well as national initiatives and policies.
- So, I will be asking you to discuss what you have experienced or consider to be exceptional care as it is currently delivered in Ireland across these levels.

**Question 1**

*First, we are going to talk about what makes a GP or general practice nurse exceptionally good. This might be that they always listen to their patients or they follow-up with patients after the consultation.*

If we think about an exceptional GP or GP nurse, what does that look like to you?

- What about an exceptional GP nurse? Are there are similarities/differences?

**Question 2**

*Now, we are going to talk about what an exceptional patient looks like. This might be that the patient always attends scheduled appointments or that they ask questions and engage with their care.*

If we think about an exceptional patient, how do they behave?

**Question 3**

*Next, we are going to talk about the how the practice staff work together as a team to provide exceptional patient care. By team, we mean GPs, practice nurses, and admin who work together as a team on a daily basis at the practice. This might be that the team members have good relationships with one another or that they all feel like they are equal.*

If we think about an exceptional practice team, how do they work together?

**Question 4**

*Now we are going to talk about the role of the practice environment or practice management in providing exceptional patient care. So, it might be that these GP practices use prompts to remind patients of well-care visits or have management who support staff training.*

If we think about an exceptional practice, how does it function?

**Question 5**

*Next, we are going to think about what exceptional care looks like when the patient accesses or is referred for further care outside of the GP practice, as well as any other factors external to the practice that impact care provision. This could be that speciality services, such as pharmacy, are located beside the practice, or that patients receive quick referrals to specialist care.*

If we think about exceptional care in terms of the interface between the GP practice and other healthcare services (e.g., hospital care), what does that look like to you?
