# Supplementary material for: Strategies that facilitate the delivery of exceptionally good patient care in general practice: a qualitative study with patients and primary care professionals
Source: BMC Prim Care. 2024 Apr 27;25:141. doi: 10.1186/s12875-024-02352-1 (PMC11055247; doi:10.1186/s12875-024-02352-1)
Supplement: Supplementary file 3 — Supplementary Material 3 [file 12875_2024_2352_MOESM3_ESM.docx]

**Additional Table 1.**

*Comprehensive overview of factors, subfactors and strategies that support the delivery of exceptional care in general practice*

| **Category (%^a^)** | **Subcategory (%^a^)** | **Strategies (%^a^)** | **Exemplar quotes^b^** |
| --- | --- | --- | --- |
| **Patient- Q2. If we think about an exceptional patient, how do they behave?** | | | |
| **Empowerment and advocacy (91%)** | Feels empowered as a patient (64%) | - Demonstrate agency and ownership over own healthcare (e.g., following up on results, bringing advocate to consultation) (46%) | *“Exceptional patients are people who put a lot of effort into, maybe working with you, but then for themselves in managing whatever it is, is going on, so that they become partners in whatever is going on.” (GP6)* |
|  |  | - Clearly indicate their healthcare needs, preferences and expectations to the provider (33%) | *“...a good patient is able to advocate for themselves or like I said, be clear on why they are suggesting something or why they are requesting something.” (P1)* |
|  |  | - Be willing to take part in practice research and are open to having medical students present (3%) | *“...if I have a medical student sitting with me, they’re almost always happy to have that.” (GP1)* |
|  | Engages in shared decision-making and care planning (42%) | - Adhere to the agreed treatment plan as best as they can and try to make suggested lifestyle changes (21%) | *“…an ideal patient is someone who would follow up on that, who will kind of adhere to the treatment they’re receiving. (P8)”* |
|  |  | - Be willing to build a shared understanding with the provider and share decision making (27%) | *“…a willingness to undertake shared decision making and shared care, and that's, not to say that often you go through the whole thing of pros and cons of the risks and benefits of various treatments. (GP1)”* |
|  | Recognises important health issues and provides accurate and useful information (36%) | - Provide clear and useful feedback and history to the provider and actively engage in care planning (33%) | *“…they come to the consultation prepared, you know they've kind of thought out in their head, you know, what their symptoms are. (GP8)”* |
|  |  | - Differentiate between urgent and non-urgent issues (6%) | *“…an exceptional patient as well should be taught in some way to recognize when a problem as distinct from say well that pain in my chest is indigestion (P13)”* |
|  | Actively seeks health information (27%) | - Actively engage during the consultation and ask for clarification and expansion as necessary to facilitate understanding of health condition and treatment plan (24%) | *"…ask the doctor any concerns, any questions or concerns you have about the treatment, about the long term effects or about the side effects of the medication. (P5)"* |
|  |  | - Independently research relevant health information and seek the provider's input as necessary (12%) | *“I love a patient who comes in and says ‘I think a bit of this and I’ve looked at a bit of that and I’d like to discuss a bit more, and whatever else’… (GP11)”* |

| **Communication and rapport building with provider (79%)** | Builds a rapport and a supportive relationship with the provider (52%) | - Treat their provider and staff at the practice with respect and pleasantness (39%) | *“...the patient should be kind of courteous and respectful to the GP… (P1)”* |
| --- | --- | --- | --- |
|  |  | - Try to develop a rapport and a good relationship with their provider (15%) | *“…that you've built up a relationship and trust… (PN3)”* |
|  | Trusts the provider and is open and honest (48%) | - Trust in their provider's clinical expertise and judgement, and believe that they are looking out for them (33%) | *“…there is an element of trust when you’re going to see this professional, you should be willing to kind of believe their judgment or kind of follow what they’re advising... (P8)”* |
|  |  | - Communicate in an open and honest manner with their provider (30%) | *“...they're honest as well about how they're feeling and what they're struggling with and what they're feeling about what you're saying and stuff like that which I find really beneficial. (GP6)”* |
|  | Effectively communicates concerns and listens to the provider (42%) | - Give good concise articulation of concerns and how they are affecting them (33%) | *“…they’d be clear in their symptom description, and they’d be clear in how their symptoms are affecting them in their day to day… (GP5)”* |
|  |  | - Listen attentively to what the provider says in the consultation and try to remember important details (12%) | *“I’d say for a patient, listening to the GP and what they’re saying… (P14)”* |
| **Beliefs and motivation (76%)** | Has reasonable expectations around healthcare (30%) | - Are understanding of staff in the practice and if appointments are late due to emergencies (15%) | *“...the patient too has an understanding that whoever is in there is in there for a reason, it's just to have that understanding. (P13)”* |
|  |  | - Make good use of time in practice and work within constraints of appointment (e.g., choose one or two issues only to discuss) (12%) | *“I think there is respect for the GP’s time, like you know I said, my practice has a thing about no more than fifteen minutes…people do do things like to try and bring kids in and get the two seen in the one appointment, or try and cover 3 or 4 health issues. (P1)”* |
|  |  | - Recognise limitations of general practice and order tests appropriately (9%) | *“...they recognize the pressure that general practice is under in terms of accessing care and delivering care, and are mindful of that (GP1)”* |
|  | Embraces advice and guidance (30%) | - Be willing to listen to advice and suggestions from the provider and the knowledge they can impart (30%) | *“...listen to what they're saying, take on board, you know, the advice, obviously with medication, but also maybe like lifestyle things... (P4)”* |
|  |  | - Willing to try new best practice and treatment options, pharmacological or non-pharmacological (6%) | *“...somebody that's open to maybe what the best practice is, or what the guidelines are for a medical condition, treatment options be it pharmacological or non-pharmacological. (GP60)”* |
|  | Feels motivated to improve their health (27%) | - Demonstrate commitment and a personal interest in maintaining their health (18%) | *“…they're interested in their own health and wellbeing and they want to do the best. (PN4)”* |
|  |  | - Seek care when they have a significant need for healthcare (9%) | *“…they seek health care or advice when it's necessary (GP1)”* |
|  | Resiliently works to the best of their abilities (15%) | - Try to do the best they can for their care with the resources they have available (15%) | *“...the exceptional patient is the one who is working with you to the best of their abilities, whatever their abilities may be. (GP10)”* |

| **Attendance and healthcare utilization (58%)** | Turns up on time or informs the practice (42%) | - Turn up on time to their scheduled appointments or inform the practice if they will be late or need to reschedule (42%) | *“...if they can't attend the GP practice, they will actually phone the surgery and notify them that they're running late or that they have to reschedule their appointments ahead of time (GP8)”* |
| --- | --- | --- | --- |
|  | Manages own care effectively and efficiently (24%) | - Organise and attend necessary routine appointments and inform the practice if they need a longer appointment (21%) | *"...an exceptional patient would attend for their reviews when they're supposed to, attend for their blood tests when they’re supposed to… (GP5)”* |
|  |  | - Organise repeat prescriptions in a reasonable amount of time (6%) | *"...they get their medications in a timely fashion, they’re not ringing five minutes before they go to the pharmacy when they run out (GP5)”* |
|  |  | - Pay for services they receive at the practice within a reasonable amount of time (6%) | *“...they pay their bills if they have bills (PN1)”* |
| **Provider- Q1. If we think about an exceptional GP or general practice nurse, what does that look like to you?** | | | |
| **Communication and rapport building with patient (100%)** | Listens to the patient (73%) | - Give the patient their full attention and make the patient feel like they are listening and not rushing (48%) | *“…making people feel that even if you’re having a very busy day, making people feel like they’ve been listened to and that you care. (P1)”* |
|  |  | - Listen attentively to what patient is saying and give it credence (42%) | *“…that they’re listening attentively to what you're saying and what your issues are, and that they are giving it the credence that it deserves (P13)”* |
|  | Reassures the patient and alleviates their concerns (61%) | - Try to make patients feel comfortable and use techniques to put them at ease (e.g., making small talk), especially during difficult procedures (42%) | *“…maybe techniques around like kind of relieving the stress there. Like I remember a nurse was just saying ‘it’s just going to pinch"...just to make it better. (P8)”* |
|  |  | - Identify the patients' concerns and reassure them where they can (e.g., explain patients concerns definitively) (36%) | *"...you find out what it is that they’re worried about. You don’t ridicule them you address them directly and point out in as clear terms as you can logically, as to why you're pretty certain that couldn't be without sounding patronizing... (GP6)”* |
|  | Builds a supportive relationship and gets to know the patient (61%) | - Build a long-term relationship with patients and leverage it to provide necessary care (42%) | *“…we do have that continuity of relationship we do have that longevity of relationship we often have an intergenerational relationship across families, so yeah it is it's that your care goes beyond the consultation, but also beyond that typically medical (GP11)”* |
|  |  | - Get to know about the patient on a personal level and find commonalities (36%) | *“I would have worked in a rural area before, and particularly some of the older male farmer patients were kind of suspicious of me maybe or nervous coming into a young female GP, and it's actually finding something to, some commonalities. (GP60)”* |
|  | Trusts the patient and treats them with dignity and kindness (52%) | - Treat patients in a respectful, friendly and caring manner (45%) | *“The big, big thing is that they're kind and caring, I think there at the outset, if that's present, generally it is the foundation for good care. (PN4)”* |
|  |  | - Communicate in an open and honest way with the patient (9%) | *“…and the ability to be honest, if mistakes are made to own up to it and say ‘look I’m sorry, and this is what I’m going to do to put it right’. (GP13)”* |
|  |  | - Trust the patient to follow action plan as best as they can (6%) | *“The doctor has to trust you to listen to them, ultimately, and you do what they say. If they refer you to somebody, they're trusting that you're going to go and see them. (P2)”* |
|  | Communicates effectively with the patient (42%) | - Pick up on patient cues and tailor their communication style to suit the patient (24%) | *"I think it's finding a level that's appropriate for the patient. So, it's finding out what they want to know, how much they want to know, and knowing how to pitch it. (GP60)”* |
|  |  | - Demonstrate clear and direct communication to the patient and focused body language (21%) | *“I really appreciate when someone like even sits down with me and is taking their time to communicate directly with me, like face to face, their body language is focused towards me as a patient. (P10)”* |
| **Coordination of care (91%)** | Coordinates good future care and clarifies referral pathways (73%) | - Understand the referral infrastructure and make effective use of it to get patients the most appropriate services and supports (58%) | *“…trying to choose sometimes who you'd send people to, that can be a really good referral, you know with complex issues, who would manage that well and provide exceptional care. (GP12)”* |
|  |  | - Clarify different options for care (e.g., private) and describe what to expect as part of referral (24%) | *“…when people want referrals or people ask for referrals it’s useful, like here in Ireland, for the GP to, I suppose, clarify people’s options. (P1)”* |
|  | Supports continuity and comprehensive monitoring (61%) | - Return results and feedback to patients as promptly as possible and ensure end-to-end investigations (33%) | *“...there’s something really like personal as well about receiving a phone call from your actual GP or like the doctor or nurse, just like the follow up to give its results. (P4)”* |
|  |  | - Comprehensively monitor patients' conditions and develop long-term care outcomes (27%) | *“...along with that is a monitoring going on, so if you're getting your bloods done on a regular basis, it’s always checked out, that everything’s working fine. (P13)”* |
|  |  | - Contact patients to check how they are doing (e.g., with certain treatments, infrequent attenders, vulnerable patients) (24%) | *“Exceptional GP is also about not just waiting for them to come to you but… that you kind of get them to come in, or you ring them and say, ‘well, I haven't seen you in six months what's happening?’ (GP10)”* |
|  | Gathers key information about the patient (39%) | - Use consultations to ask non-medical questions about the patient's background, life and current circumstance (36%) | *“…to engage with patients around the non-clinical aspects of care, around the bits of life that actually have very real implications for care but aren’t necessarily technically medical or technically nursing. (GP11)”* |
|  |  | - Pass on important medical and non-medical information to other providers involved in the patient's care (12%) | *“…now with all the vaccinations going on, an exceptional nurses will be able to pick up on the smaller things and pass that on to a GP.” (P14)* |
|  | Prepares for and manages the consultation (36%) | - Prepare adequately for the consultation (e.g., read patient information prior to consult, prioritise by patient need) (27%) | *“…if the practitioner doesn't know the person very well, that they would have spent the time before the person comes in to have some idea of the background…” (GP3)* |
|  |  | - Elicit the patient's agenda for the consultation early and actively manage the consultation (9%) | *“…to kind of check the agenda for the beginning of the consultation. It’s important that they kind of get their own agenda out as well, so that you can plan what can be managed safely within the 15 Minutes…” (GP8)* |
| **Capacity and skills (88%)** | Manages their time effectively and is organized (58%) | - Actively manage time within the consultation while ensuring patients have enough time to safely discuss their concerns (39%) | *“…to make you feel like they've given you a certain amount of time. I know they can’t stay with you all day, but they give you a certain amount of time, they listen to your concerns and they devise a plan of action.” (P5)* |
|  |  | - Organise and structure tasks to be performed for the day (21%) | *“...to do the skills...plan them first of all, you go in in the morning and plan what you have to do, do the skills, carry them out and then follow them through.” (PN2)* |
|  | Exceeds patient expectations (48%) | - Advocate for their patients’ needs and preferences and follow up on missed care with other services (24%) | *“…advocated for them, you know, by phoning this person and that person writing this letter and that letter and just kept chasing things until you got the best outcome for them in the most timely a way as you could.” (GP6)* |
|  |  | - Give patients more time in difficult consultations, and offer to see certain patients more often (21%) | *“Often with kindness people take an extra step, they give more time to patients or they go out of their way to make a phone call to see how the patient is after a consultation with it, or they may offer to see them more frequently.” (GP12)* |
|  |  | - Go the extra mile to get patients the best care they possibly can and make sure they are prepared for their care plan (e.g., fitting patients into schedule, writing down information) (18%) | *“…your patient might be worrying about something else, but it might be going the extra mile to try and make that person's life some bit better, be it physical, psychological, socially.” (GP60)* |
|  | Knows their strengths and limitations (39%) | - Work within your scope of practice and know when to escalate problems and seek second opinions (27%) | *“…you need to be able to know at what point do I need to go and get that GP for something but also you really need to know like, somebody who's really competent is able to make a judgment call, but that is within their competence level.” (PM2)* |
|  |  | - Work to strengths and willing to admit and address weakness (15%) | *“I just let it go really and focus on other things, so it's recognizing your strengths and your weaknesses and trying to address them as best one can but equally being pragmatic and using the strengths of other people in the practice team.” (GP1)* |
|  | Is skilled in problem solving and decision-making (33%) | - Be comfortable and efficient at making decisions (18%) | *“…you spend your whole days making decisions, we carry an awful lot of risk and you really have to be comfortable with that.” (GP10)* |
|  |  | - Be creative and pragmatic, and use available resources efficiently to avoid waste (12%) | *“A really good GP is bit more, it's bit less prescriptive, a bit more creative in that way…it's kind of more having to invent ways around things that otherwise might be impossible.” (GP6)* |
|  |  | - Be able to problem solve independently (9%) | *“I think that's really good judgment there and being able to problem solve on your own, but not being such a crusader, that you know when it’s the right time to work as a team.” (PM2)* |
|  | Engages in risk management (24%) | - Conduct an appropriate level of investigations, and diagnose anything dangerous early and deal with it appropriately (15%) | *“...the exceptional GP or GP nurse what they're able to do is kind of pick out the headache that's a real headache that really needs an MRI and needs to be sorted versus maybe the headache that might have other things going on, so they've got both kind of antennas up at the one time...” (PN3)* |
|  |  | - Pay attention to safety protocols and risk management, and keep on top of admin tasks (12%) | *“...it's really just attention to protocol...there is a following of evidence and of appropriate ways of doing things.” (GP6)* |
|  |  | - Engage in safety netting and leave the door open for patients to return if the issue persists or worsens (12%) | *“…communication is a huge part of that, both acknowledging that uncertainty and providing the safety nets around it, that patients are still safe even when the diagnosis isn't clear.” (GP12)* |
| **Beliefs, attitudes and motivation (85%)** | Takes a holistic approach to health and healthcare (48%) | - Treat the 'whole person', and consider the patient's personal context (e.g., work, social environment), family history and background in decision-making (30%) | *“…they know a person, they know their whole family, they know the social environment they live in, they know their job, they know their income, they know all these things, you know, and really, all the time are kind of considering them.” (GP9)* |
|  |  | - Look past the presenting symptoms to try and uncover the cause (21%) | *“…exceptional care in my opinion would include looking for the cause, be it stress, be it lifestyle, be it diet, and relating that to the symptoms that are being displayed.” (P13)* |
|  |  | - Prescribe both medical and non-medical treatments to patients (e.g., social prescribing) (15%) | *“…referring people to our social prescriber is really great...and then if a psychological intervention is necessary as well, but often social support helps bolster our psychological capacity to cope with events, with life.” (GP9)* |
|  | Is non-judgemental and compassionate (45%) | - Accept the patient without judgement and try to make decisions unbiased by their background, history, age, gender or other patient factors (33%) | *“I think that really helps with exceptional care as well, is that all accepting and non-judgmental, and recognize each other, as you know, humans with the same common universal experience of life in general.” (GP9)* |
|  |  | - Understand the role of social determinants of health and treat patients with compassion and an understanding (30%) | *“…they're a bit more with it with the research, like the new research on kind of social determinants of health. Not just like a model that it's patients’ personal responsibility if they're sick, they’re also considering poverty and racial inequality, things like that.” (P10)* |
|  | Is motivated to provide care (39%) | - Demonstrate a genuine interest in patients and are suited to proving care in general practice (27%) | *“…committed, they're all kind of the obvious things but you know, that they're passionate about their job, passionate about general practice.” (GP10)* |
|  |  | - Be persistent in providing important care to patients and capitalise on every opportunity to give patients important advice or treatments (18%) | *“…very good at capitalising at these opportunities where they have people in to, I don’t know, maybe if I smoked they tell me to stop smoking, they kind of check health behaviours.” (P1)* |
|  | Values patient's perspective and respects needs (33%) | - Respect the patient's understanding of their health and healthcare preferences (e.g., use of safe non-medical treatments) (21%) | *“…it’s really not dismissing the patient’s concerns offhand, because patients kind of know themselves what they are experiencing, so I think it’s really respecting the contribution the patient can make to understanding their own health.” (P1)* |
|  |  | - Focus on treating the person and not the illness (12%) | *“I think the fundamental job of a GP is to be patient-centred, which means that your agenda is not about the disease, it's about the person who has the disease…” (GP6)* |
|  |  | - Respect patients' privacy and maintain confidentiality both inside and outside of the consultation (6%) | *“…there’s an element of discretion, dealing with people, a large element of discretionary really.” (PN1)* |
| **Patient activation and education (76%)** | Educates patients and encourages shared decision-making (36%) | - Identify and take into consideration what the patient wants and encourage the patient's involvement in clinical decision-making (27%) | *“…giving choices, making suggestions and helping people to choose what suits them, in their particularly life, I think, is a really important part.” (GP9)* |
|  |  | - Provide information to patients on their condition and unbiased reputable information on different treatment options (18%) | *“I find with my own GP, if I’m put on a medication, like a longer term medication, which I was a few years ago, he explained the side effects really well…” (P11)* |
|  | Supports behaviour change (33%) | - Provide practical advice and encouragement on changing behaviours (24%) | *“My doctor would be really good at looking at like how I can change it myself, to help better my condition myself, so I think that’s really good, just trying to see how they can help the patient change.” (P4)* |
|  |  | - Ask the patient about health behaviours during routine consultations and investigate the context around behaviours (9%) | *“They do the Make Every Contact Count thing, that’s something an exceptional GP should do!” (P8)* |
|  |  | - Allow the patient to set their own behaviour change goals (3%) | *“I’d be very much like ‘what do you think, what's your first goal, what would be if you could change anything about your life, what's the first thing you’d change?’ (PN3)* |
|  | Engages patients as partner in care (30%) | - Try to enhance patients’ sense of agency and empowerment over their health and condition, particularly patients that are hard to reach (18%) | *“...a really, really good GP will always say to the person, you know precisely what to watch out for without frightening them, so if this happens that happens, I need to hear about it.” (GP6)* |
|  |  | - Centre the consultation on the patient and involve them in decision-making from the consultation to follow-up (15%) | *“...that you're kind of kept in the loop as well. I think a lot of the time, decisions can be made or kind of actions can be taken, and you don't really know why…” (P8)* |
|  | Proactively manages and prevents illness (15%) | - Monitor chronic illness in the practice and help patients manage their illness and treatment plan (12%) | *“…you manage the chest infection but then get the nurse to follow up with them on the care management of their asthma and look at their inhaler technique…talk to them about their triggers, what might have led to this and how they could prevent it in the future.” (GP9)* |
|  |  | - Ask patients about and try to engage them in important preventative care interventions (e.g., flu vaccines, smear test) (6%) | *“…the GP nurses are quite proactive in trying to engage people in preventative care, so like ‘have you checked your smear tests’, you know ‘have you done x or y’, or you know ‘are you doing this.’” (P1)* |
| **Continuing Professional development (33%)** | Self-reflects and wants to improve (21%) | - Show interest in learning new things and developing specialisations as a provider (18%) | *“...it's kind of just giving people the opportunity to develop areas of interest and encouraging that within the practice.” (GP13)* |
|  |  | - Reflect on their own practice and look at how to improve, occasionally taking part in research (6%) | *“...an exceptional GP would constitute somebody who is constantly looking to improve.” (P14)* |
|  | Keeps up to date with best practice (18%) | - Independently keep themselves up to date with new protocols and standards, and evidence-based practice (18%) | *“To remain up to date on the protocols and procedures on what you're doing, and that you educate yourself appropriately as it goes on…” (PN1)* |
|  | Attends formal education (12%) | - Attend continuing medical education schemes and take formal courses in areas of specialty (12%) | *“We're all part of a CME [Continuing Medical Education] group, we have a local CME tutor and everybody is part of a CME group.” (GP9)* |
| **Clinical Microsystem- Q3. If we think about an exceptional practice team, how do they work together?** | | | |
| **Team collaboration (97%)** | Collaborates to provide integrated care (70%) | - Coordinate effectively to provide seamless services for patients (e.g., allow admin staff to pass on messages, review results as a team) (58%) | *“…even like little things like when you come out the payment is prompt, or they’ve been informed by the GP exactly what kind of consultation you had, so that's all very quick.” (P1)* |
|  |  | - Refer patients within the team and help out colleagues to provide integrated care to patients (e.g., ask questions) (27%) | *“…they've got the support of their colleagues so if something is, and they just need a second opinion or they're not 100% confident with it, that you've got that inter-colleague relationship that you can really confer with one another and things like that.” (PN2)* |
|  | Uses structured lines of communication (67%) | - Maintain open methods to communicate, debrief and share patient information regularly amongst the team (e.g., whiteboards) (42%) | *“…it's keeping that line of communication open, so I think there's that element of how we communicate issues or problems or suggestions, even having communication boards like whiteboards in the staff room.” (GP60)* |
|  |  | - Ensure communication about patients amongst the team remains respectful and discreet (27%) | *“…another point with that, is their means of communication. Like it's so important to have really efficient and direct means of communication between, say, the doctors, consultation rooms, reception, but, I also think it's very important for it to be discreet as well. (P4)* |
|  |  | - Use technology to support communication and teamworking (e.g., daily electronic tasks, electronic messaging applications) (21%) | *“…often the only way we communicate if we're not seeing each other might be on that computer system. So, if we've spoken to the patient, if we've done a task… that is inserted on the system so that the next person who comes in can pick it up clearly.” (GP60)* |
|  | Has regular practice meetings (55%) | - Operate regular practice meetings to ensure good management of the practice, with more regular meetings where issues arise (52%) | *“So, kind of weekly meetings, monthly meetings, they have to be listened to, you have to feedback, and vice versa.” (GP4)* |
|  |  | - Ensure engagement with meeting and communication of discussions from meetings to all staff (e.g., full team or parts of the team are present, provide minutes of meeting) (21%) | *“…then feeding back as well from the outcome of the meeting, there's always a minutes of the meeting sent to everybody.” (GP60)* |
| **Team efficacy (94%)** | Values and trusts team members (85%) | - Value and respect the contributions of every team member (e.g., value admin role in managing workload, triaging patients) (82%) | *“I think it's really important than that you know every staff member feels valued, so they know their work is really important.” (PN3)* |
|  |  | - Have a teamwork orientation and trust other team members to complete tasks and execute their role effectively (24%) | *“I would feel we have a core of very good, excellent colleagues that I really trust, that I just absolutely trust. If I need them to do something I absolutely trust that they’ll do that and they're exceptionally good at doing it.” (GP2)* |
|  | Understands team roles and needs (55%) | - Ensure that every team member’s role and responsibilities are clarified, and that everyone knows how to execute their role successfully (33%) | *“I think having well defined roles as well where possible...knowing your role, insofar as in these are the tasks I would usually perform but being open to helping in other areas, and being flexible as well.” (GP60)* |
|  |  | - Discuss and develop a shared understanding of every team member’s role, and the needs and challenges of the team (27%) | *“I will sit our reg [registrar] on the phones for at least half an hour to an hour on any day just to gain an understanding, and again to understand all your team roles, to gain an understanding of what is happening at the front before it even gets to you.” (PM1)* |
|  | Shares tasks and harnesses team strengths (42%) | - Share tasks and workloads appropriately, and be willing to share workloads with others who are overburdened (27%) | *“…a willingness to not pick up the slack but you have to be willing to share the workload with people a lot of the time.” (PM2)* |
|  |  | - Delegate tasks and roles to the most appropriate person, often based on team strengths and weaknesses (24%) | *“…everyone finds their strengths, and they play to their strengths in the team and it works out then and just appreciating those qualities of the different members, it really enhances it all.” (PN4)* |
|  | Has a multidisciplinary structure (27%) | - Involve everyone in the practice as part of the team, including the patient, advocates, and cleaners (15%) | *“…if you're in care of someone, for example, while there's no breach of confidentiality, that you're caring of someone is acknowledged by the practice…that you're part of that team as well.” (P13)* |
|  |  | - Have providers with different qualities, qualifications and specialisations (e.g., nurse specialised in diabetes care) (12%) | *“Nurse prescribers would be a big help...there is a small cohort of stuff they prescribe, and that would really help in GP land, and then there's the advanced nurse practitioner, who would actually take over a whole cohort.” (PN1)* |
| **Team rapport (88%)** | Creates a supportive and friendly working atmosphere (67%) | - Foster a happy supportive working environment where everyone is treated in a kind and friendly manner and people are open to having a laugh (42%) | *“…that whole friendly and relaxed atmosphere, I think it's really important. You notice that straight away…like friendly, relaxed and then just, you can kind of tell, you know, people are smiling, people are happy in their work.” (P4)* |
|  |  | - Offer each other emotional support and look out for and support those who are struggling (36%) | *“It’s more important, I think, for the team to know that you literally have the support system there for each other.” (PM1)* |
|  | Builds and maintains good relationships (45%) | - Get to know each other on a personal level and try to develop good relationships (27%) | *“I think there can be the informal interpersonal relationships, where you have dinner together, you have food together, you have a laugh together, you might know personal details about each other…” (PN2)* |
|  |  | - Get on well with each other and resolve interpersonal issues pre-emptively (24%) | *“…where I go to my doctor, that they seem to gel very well, so like from the receptionist, to the nurse, to the doctors, they all seem to go get on very well together.” (P6)* |
|  | Has opportunities to meet informally (36%) | - Organise dinners and social meet-ups yearly as well as for special occasions (e.g., new staff member joining the practice) (24%) | *“So, that's very simple things like every year, we have Christmas party where we all go out and bond...we try and go out for lunch if there’s a success, somebody is 50…we try and just have a lunch together, whether it's in the practice or whether its downtown...” (GP4)* |
|  |  | - Meet regularly for lunch and coffee, and allow the team time to bond over non-medical tasks (e.g., picking a colour scheme for practice) (18%) | *“…we have a half an hour every day where we all sit around as a team, the admin, the GPs, the nurses, and we just from two to half two, we have a cup of coffee or tea and lunch and we just have a chat.” (GP13)* |
|  | Treats one another with dignity (33%) | - Treat each other with dignity and respect, and talk about other colleagues respectfully (27%) | *“Well anytime that I’ve ever seen a doctor talking to a receptionist or nurse they always talk to them with respect.” (P6)* |
|  |  | - Be open and honest when communicating with one another (6%) | *“…you just need that sense that people can be honest and straight with each other in clinical care settings…” (GP6)* |
| **Patient-focused culture (58%)** | Makes the patient feel welcome and valued (33%) | - Treat patients with friendliness and respond in a manner suited to the patient (24%) | *“…such kind people to the patients but also, just really, really intuitive about how to respond to the patients.” (GP2)* |
|  |  | - Make the patient feel important, involved and valued as part of their team (21%) | *“…in terms of people who may be in the room with the patient… that they communicate with each other about the patient, but to make the patient feel involved.” (P2)* |
|  | Respects and advocates for patients (36%) | - Treat patients with respect and understanding from making appointment until follow-up, and investigate complaints with respect for both sides (27%) | *“…from setting up the appointment until you're paying on your way out, that the whole thing is something very kind of respectful.” (P3)* |
|  |  | - Show patients that the team have a plan of action to support them (9%) | *“…that you come home feeling, well there’s a plan of action in place, so it's not just me dealing with this on my own, there’s a team out there that can bring you through whatever is going on.” (P5)* |
|  |  | - Identify and accommodate vulnerable patients (e.g., anxious patients entering the practice) (9%) | *“...somebody could be coming in, anxious and maybe very hyper, talking very fasts...that they would have the techniques and the way to learn how to make sure you know, settle that person down a little bit, tell them where the waiting room is…” (P3)* |
| **Learning culture (42%)** | Learns from when things go wrong (27%) | - Foster a no blame culture where people feel comfortable reporting errors and support each other when things go wrong (24%) | *“Like on a daily basis, I would say some or all of us are having one of those chats and you know if something doesn't go right, it isn't a culture of blame, it’s a culture of learning and discussion.” (GP11)* |
|  |  | - Learn from cases, incidents and errors together as a team to mitigate future risk (18%) | *“…we met once a month, we had an educational topic but then we had some just time to chat and to reflect on something and to throw out something that happened… you know how would I manage that better the next time and things like that...” (PN3)* |
|  | Embraces change and reflects (21%) | - Demonstrate good problem sensing as a team, noticing and discussing issues pre-emptively and ensuring systems are in place to improve (24%) | *“…being responsive to problems, identifying when there are problems and then working to fix them instead of just sort of sticking your head and hoping that it will sort itself out, you know, waiting for the crisis to hit, trying to be pre-emptive about things.” (GP10)* |
|  |  | - Demonstrate responsivity, flexibility and a willingness to change (15%) | *“…being open to change is important, if we get stressed, we get rigid and we’re not happy to take on new ways of practicing, and it changes all the time…things are changing and nothing is static, we have to adapt to what research is saying and what needs are.” (GP9)* |
|  | Dedicates time for learning opportunities (9%) | - Schedule emergency meetings for serious events and allow for staff to debrief after an incident has occurred (6%) | *“…when we all got off the phone and went ‘Jesus Christ what just happened’, is that immediately our practice manager phoned the patients who'd been delayed...so anything that was non urgent got rescheduled, freed up some time and we sat down as a team and we actually had a conversation about what had gone on.” (GP11)* |
|  |  | - Conduct robust investigations of critical incidents, errors and near misses that have occurred (6%) | *“…if there's a near miss, that it’s flagged, that you go back to the person and people involved, find out what happened…That you're robust about dealing with that sort of stuff…why did that happen, what does that mean for the practice, what do we need to change, does everyone know what should have happened and what went wrong?”(GP10)* |
| **Mesosystem- Q4. If we think about an exceptional practice, how does it function?** | | | |
| **Facilities and infrastructure (100%)** | Provides a pleasant and safe environment for staff and patients (91%) | - Maintain a well-organised and up-to-date bright, spacious and aesthetically pleasing environment (e.g., plants, artwork) that is conducive to patient flow and allows for flexibility in room organisation (79%) | *“…it's modern, it's quite a nice building…it's very spacious, airy, feels very clean, no carpets, everything, surfaces clean, clean, clean.” (P7)* |
|  |  | - Try to create a comfortable environment for patients (e.g., water, comfortable seating, TV or toys for kids) with adequate bathroom and baby changing facilities and potential for a separate waiting room (48%) | *“An exceptional practice would be set up to meet the needs of the patient, so you know a water dispenser, appropriate seating, and comfortable seating for the different needs of patients.” (GP60)* |
|  |  | - Create an environment suited to staff needs, including a comfortable work space, staff room and cafeteria, and bathroom facilities (18%) | *“A coffee room, a social space of some sort where the staff can actually not be at their workplace, and if the opportunity to socialize briefly presents itself, all the better. Adequate toilets, I mean I know it sounds obvious, with baby changing facilities.” (GP11)* |
|  | Uses IT systems effectively to support the delivery of care (79%) | - Make effective use of different functionalities of the system to optimize care and provide feedback on the tool as necessary (e.g., recall, safety popups, audit, tracking features) (67%) | *“...there are also add-on things to the basic package in terms of online appointment booking systems, online payment systems and like a practice management suite….tracking how many patients came for this, how many patients came for that, the recall systems in terms of people due to come back again for cervical tests or vaccinations…” (GP3)* |
|  |  | - Provide a practice website that allows for online booking of appointments, ordering of prescriptions and payment (30%) | *“What could be handy as well, is that on like a GP’s website to have information about what GPs are there, whose employed and their names.” (P8)* |
|  |  | - Use technology to communicate with patients and further care services (e.g., text service, electronic prescriptions, electronic referrals) (18%) | *“…we have utilized our text messaging system like beyond belief in the last two years, we use it to communicate normal test results and things like that to patients, and we also have used it incessantly for COVID vaccines and everything like that.” (PM2)* |
|  | Provides parking facilities and good accessibility (52%) | - Provide good and safe access to the practice, with a wheelchair ramp, lift and other methods of ensuring good accessibility for different needs (39%) | *“…accessibility would be number one, so an awareness of the range of people that are likely to be coming into the practice. Very basically, to be wheelchair friendly, so not just wheelchair accessible, but wheelchair friendly, and having consideration for all of the different types of people that might be coming in.” (GP3)* |
|  |  | - Provide clearly marked parking facilities for patients and staff (30%) | *“…there's a good and a clear space to park, clear instructions, like this is for, you know, the GP.” (P4)* |
|  | Has necessary equipment and resources (39%) | - Ensure all necessary equipment is available and easily accessible within the consultation room or practice (30%) | *“…the room should all be well stocked, have the necessary equipment, if you’re doing bloods, the necessary bloods, the forms.” (GP13)* |
|  |  | - Provide comprehensive diagnostic facilities at the practice for bloods, echocardiography, electrocardiogram and other tests (12%) | *“…having the facilities nearby, within the practice for bloods or ECHO sounding, ECG, all of those type of things, rather than been referred to as here, to [the emergency department in the city].” (P13)* |
| **Appointments and access (88%)** | Has a careful system for scheduling appointment (48%) | - Have a highly organised system for scheduling appointments, that balances continuity of care for chronic disease versus those acutely unwell (e.g., keeping emergency slots open per day) (36%) | *“…we allow appointments to be booked up months in advance but equally we keep a certain number of appointments for people who are sick on the day. So it's trying to balance the long term needs of people and convenience in terms of making an appointment when it's convenient for them.” (GP1)* |
|  |  | - Try to support continuity by allowing patients to visit the same GP or nurse when they attend (15%) | *“Continuity, well continuity is difficult at the moment with the staffing issues, continuity with the GP.” (PN1)* |
|  |  | - Operate a cancellation list and provide a way for patients to cancel appointments easily (6%) | *“…we have a dedicated text cancel line so people can literally just text to cancel the appointment as opposed to trying to get through on the phone.” (PM1)* |
|  | Actively facilitates access to care (48%) | - Send reminders of appointments to patients, with important information if necessary (27%) | *“Definitely prompts and reminders of bookings, even maybe details, if it was like important to fast or something like that beforehand, or whatever was required before the visit.” (P8)* |
|  |  | - Make it clear to patients how to check in to the practice, using signage or by directing people (15%) | *“…clear, very clear instructions on maybe like the check in process.” (P4)* |
|  |  | - Provide some flexibility in the timing of appointments where possible (e.g., squeezing in a patient just before lunch) (12%) | *“…they would facilitate if you're working, your work life, and so maybe early morning ones or late afternoon ones or something like that, but that there is a facility there to work around that.” (P3)* |
|  | Provides timely appointments led by patient need (27%) | - Provide appointments for patient-led issues that are non-acute in a reasonable amount of time (21%) | *“…most of the time it’s what has the patient decided they need to attend about, and that’s the most important facet of it, that accessibility and patient-led demand is really important too.” (GP10)* |
|  |  | - Provide appointments fairly and appropriately to their patient cohort (e.g., additional COVID-related appointments during the pandemic) (12%) | *“I suppose an appointment system needs to be fair, where it offers appointments to people across all different groups, whether they’re private patients or medical card holders, whether young or old.” (GP2)* |
|  | Provides different ways to book appointments and access the practice (36%) | - Provide additional means of contacting the practice and making appointments (e.g., email service, online booking of appointments) (21%) | *“If you could have like an online booking system would be great, you know, to see available appointments and that.” (P8)* |
|  |  | - Make sure that lines of communication are always open to patients so they can always get in contact with practice in some manner (21%) | *“I suppose, patients getting access to the practice whether it’s email, telephones or walking in.” (GP4)* |
|  | Triages appointments so patients in need always seen (33%) | - Triage patients to identify those with serious need and prioritise in line with patient need (21%) | *“…certain people don't need to be seen straight away, but certain people do, and that they prioritize patients with whatever's going on, that they're prioritized and, you know, need to be seen straightaway.” (P5)* |
|  |  | - Squeeze in those who need immediate care, including children and elderly patients (18%) | *“Appointments wise, I think never refusing something urgent, we’d never refuse a child, and having the scope to manage what is urgent, by comparison to what is routine.” (PM1)* |
| **Clinical management (88%)** | Utilises robust systems for clinical management (67%) | - Use technology effectively to support care systems (e.g., electronic referrals, prescriptions, and discharges) (36%) | *“…that you're able to do e-referrals with the hospital and there’s this constant communication through the practice software of information coming back from the hospital, whether it's results or appointment dates, appointment DNAs, outpatient letters...” (GP8)* |
|  |  | - Maintain efficient and prompt systems for communicating with patients about care or following up to communicate results (24%) | *“…a massive thing is the way that results are sent to patients, so blood test results or any kind of results. I think an excellent practice contacts the patients, doesn't wait for the patients to contact them about results, no matter what kind of results they are.” (P10)* |
|  |  | - Maintain robust systems for managing common care processes that are clear to staff and patients (e.g., progress of referrals) (24%) | *“…it's very important that they see what works for them. And you know dealing with phone lines, dealing with the prescriptions, like orders, blood, everything. You know lots of things going on, so important to get a good system that works for everyone…” (P4)* |
|  | Implements and adheres to standardised care protocols (52%) | - Make sure that patients and staff are aware of protocols, and what is expected of them (e.g., for checking results, making appointments) (42%) | *“…call out people when they're not following the protocols, following the guidelines in a way that's effective so that change is brought in, but also that it’s appropriate from the point of view of that it's not harming to the team.” (GP60)* |
|  |  | - Implement standardised protocols to support care processes and adhere to public health guidance (e.g., COVID protocols), that can be adapted by staff if necessary (27%) | *“…I think clear protocols, if there are particular ways that a practice wants particular things managed so for example, following up on investigations, you know…” (GP11)* |
|  | Dedicates time to providing high-quality care (12%) | - Allow sufficient time for patient consultations to be delivered safely and effectively (9%) | *“…many practices have moved to 15 minute consultations, because when you give more time, you're better able to deal with what's presenting, and then also help address other things that might have led to that.” (GP9)* |
|  |  | - Ensure time is allocated to allow partners to address management or admin issues alongside patient care (6%) | *“…ideally, it's good that the partners have time then dedicated towards more management or admin issues, that it's not something that they are doing at the end of the day.” (GP8)* |
| **Considering patient need (73%)** | Safeguards patients’ privacy (39%) | - Create private area for patients to communicate with reception and ensure communication with or about patients is discreet (e.g., when triaging on phone, directing patients to rooms) (24%) | *“…sometimes you need where people can have a private word with the secretary, we shut it back down and we actually created a little anti-room with the Perspex and the glass kind of desk to talk to.” (PN3)* |
|  |  | - Maintain the patient's right to privacy while in the practice (e.g., music playing in hallways, curtain for privacy) (15%) | *“…there's maybe music or something playing out in the general hallways so that there's no chance of being overheard in the room next door or that type of thing, so just being mindful of those type of things.” (PN3)* |
|  |  | - Ensure patient information is maintained in line with GDPR (e.g., eliciting permission for email, managing patient information) (9%) | *“…since the GDPR update he has gotten all patients to sign a consent form for their data and holding their data.” (GP9)* |
|  | Supports access for complex and hard to reach patients (36%) | - Identify and target efforts at high-risk patients, such as developing registers of high-risk patients or informing patients with chronic diseases about flu clinics (24%) | *“…we know when you're going to be bringing people in for CDMs, that there’s registers of the patients over 70 with chronic diseases, there’s a register of the diabetic patients within the practice, and they have an up to date registry of who needs the flu vaccine, who needs boosters for COVID vaccine.” (GP13)* |
|  |  | - Cater to complex patient needs as much as possible & make exceptions where possible (e.g., give longer appointments, allow in-person appointment-making for elderly patients) (12%) | *“…so important that you facilitate people who might not be able to strictly follow your practice’s policies.” (PN3)* |
|  |  | - Support and advocate for the patient from making appointment until follow-up (e.g., following up with the hospital) (9%) | *“Equally then, is busy chasing up stuff that's missing from the hospital or chasing up missed appointments or chasing up on, you know, services that patients are not getting and should be getting…So you're always batting for the patient, being accessible to the patient.” (GP6)* |
|  | Constantly considers the patient (27%) | - Create an inclusive and safe environment for all (e.g., children) (21%) | *“…we give stickers, I think, and you try and make a child friendly environment if you’re dealing with a lot of children.” (PN3)* |
|  |  | - Understand your cohort and offer services accordingly (e.g., when providing digital resources, what level of information) (9%) | *“…you're hoping that they're digitally savvy, you know…obviously, younger people will and some of the older people are great as well…But you're constantly thinking, you thinking does this suit that person, does that suit this person, there's a lot to it, actually.” (PN1)* |
|  | Provides patient education resources (12%) | - Gather and share relevant useful patient resources, technologies and leaflets that are appropriate for different patients' needs (9%) | *“…it’s important to have, you know, information available there, and also like a lot of information that you can take with you in the leaflet form, or flyers, or the business cards...” (P4)* |
|  |  | - Provide relevant health promotion information on posters, screens and/or monitors within the practice (6%) | *“…an information screen or whether they, again posters.... reminding them of the national screening programs, I suppose, taking care of their health, reminding them to have annual visits over a certain age or you know, the men's health, the women's health…to say what’s in the practice, to know what they provide in the practice.” (PN4)* |
| **Business management (67%)** | Maximises scheduling and proactively manages wait times (45%) | - Maximise scheduling and allocate an appropriate number of appointments each day so that each patient can be sufficiently engaged (33%) | *“…it's really important to be constantly checking that and looking ahead, and seeing how can we really kind of maximize the diary and get the best use of time.” (GP8)* |
|  |  | - Be proactive in managing patient flow and investigate if wait times are consistently delayed (24%) | *“It’s really important that the practice has a look at why their wait times are running over and do they need to make changes, do they need to schedule less consultations, do they need to clarify with patients, do they need to do longer consultations for certain things.” (P1)* |
|  | Has highly organised practice management structures (36%) | - Develop highly organised management structures and systems in the practice, including a practice manager (33%) | *“…it's a really good practice manager supported by a really good admin team, who all have very clear roles and responsibilities.” (GP1)* |
|  |  | - Appoint admin in charge of the logistics of practice so providers can focus their time on delivering care (9%) | *“…if you have a really strong manager who's very good at running the practice and allows you not to worry about that and get on with your job, seeing patients and not being distracted by logistics of it…” (P9)* |
|  | Sets standards and plans extensively (18%) | - Set standards for the practice to achieve and set expectations with new staff (e.g., giving new staff a document outlining role expectations) (15%) | *“…what way would we like for this practice to be, what are the things that we want to offer or to be good at, what are the things that we're not here for, what are the things that we're not going to be involved in.” (GP3)* |
|  |  | - Plan extensively for new services and programmes and demonstrate strategic direction (e.g., setting goals for the practice) (9%) | *“…it sets goals for itself, strategic direction, not just turning up day to day to do the work.” (GP6)* |
|  | Manages payment systems (9%) | - Manage practice finances and payment systems carefully and offer user-friendly methods of payment (9%) | *“...from a practice management point of view, things like managing finances, managing unpaid patients, you know, the practice cannot survive, the sustainability, the viability of the practice depends on minute detailed management of things like finances, staffing. (PM2)* |
| **Human resources management (64%)** | Effectively manages staff (55%) | - Delegate and rotate responsibilities, tasks and leadership roles to the most appropriate staff members (30%) | *“…it's really important that there is a leader, and you know, it's really important that the leader trusts their staff with other leadership roles…they’re really good at giving responsibility to other members of staff to lead on different aspects.” (PN3)* |
|  |  | - Schedule staff clearly and appropriately to allow for flexibility and ensure staff contracts are maintained (appropriate holidays, pay) (27%) | *“From the point of view of the management, I think clarity of who's on, when, what the appointment times are, and that the rotates are clear, that everyone knows who's on, and when they’re on, with regard to both medical and administration staff.” (GP5)* |
|  | Appreciates staff and supports well-being (42%) | - Verbally and overtly show staff they are appreciated from top down (e.g., days off, highlighting it on noticeboards, staff socials) (30%) | *“…it shows that it values by giving time to people and acknowledging that, and maybe publicly acknowledging that within the practice as well. That can be either explicitly by noticeboards, at meetings highlighting it, financially, or implicitly.” (GP12)* |
|  |  | - Proactively maintain and support staff wellbeing (e.g., no tolerance for abuse policy, designated wellbeing liaison) (21%) | *“…one of the really, really crucial things, we have a no tolerance policy, and I know some practices say it, but we really have it…we won't accept anybody to either come in and be abusive to, and it’s usually the front line people who get it.” (PN3)* |
|  | Provides staff induction and training (30%) | - Support providers and administrators to keep training and knowledge up to date and to develop and maintain areas of interest (24%) | *“She's now trying to get a workshop on menopause for me, because she knows I’m interested in that area, so you know she’ll send me a quick task saying you know ‘I read this if you're interested in looking at this document’.” (PN2)* |
|  |  | - Provide orientation materials and induction training to new staff (9%) | *“Even simple things, like when a new person comes to join the practice, that there is like a booklet that they are given, this is how we do things, this is how we write our notes, this is how we make referrals…so having resources for staff that they can use.” (GP10)* |
| **Innovation and improvement (52%)** | Elicits staff and patient feedback and innovations (33%) | - Use proactive methods in the practice to elicit patient and staff suggestions or complaints, such as questionnaires or suggestion boxes (30%) | *“…getting patients’ views and feedback and things like that, and you know, like we would always have a patient comment system available for people to post in, email in, drop in…” (PN3)* |
|  |  | - Try to implement patient and staff input and explain if solutions are not possible (12%) | *“…as a practice manager if there's any complaints, you know, addressing them early, having a complaint policy in place to prevent it go any further as well.” (GP13)* |
|  | Continuously monitors performance to improve care (24%) | - Respond pre-emptively to changes and continuously review, monitor and adapt systems and ways of working to improve care (21%) | *“…if they're telling you about a reoccurring problem that keeps on happening, you know, not making them change to suit it, but finding a way of changing the system so that that problem isn't arising.” (GP3)* |
|  |  | - Conduct audits and review performance at aggregate level and use own data to inform improvements (12%) | *“…they work collaboratively to review their performance, review what's going on, you know at aggregate data, not just individual care and try to get a sense of how they can all continue to improve and gain and grow.” (GP6)* |
|  |  | - Look for external guidance and research, and engage in benchmarking and sharing innovations (6%) | *“…what's really important is that we have to start publishing it more, you know, we’ve got some brilliant journals...we need to be putting a lot of information in there and encouraging practices and learning from one another with it.” (PN3)* |
|  | Promotes role modelling (15%) | - Support staff in leadership roles that take on new projects and get everyone on board (9%) | *“…as a leader, you want to, you know, take a new project, somebody that's enthusiastic, take on a new project and just kind of drive it, but you have to bring everybody with you, you have to lead from behind than the front, isn’t that what they say.” (GP4)* |
|  |  | - Support clinical role models in the practice who strive for high-quality patient care and set this expectation from the top down (6%) | *“…to lead by example…that there's an example of high-quality patient care by the leader in the practice, so that the patient is very important and that we strive to treat with compassion and also the highest quality from a medical point of view.” (GP5)* |
|  | Engaging in research and education (9%) | - Engage in teaching medical students and registrars, in the practice and/or university (6%) | *“...we have medical students and we teach the registrars, the GP registrars.” (GP4)* |
|  |  | - Take part in external research projects (6%) | *“…open to doing research, you know…it's open, engaged, curious, you know, introspective, but also open to other ideas and perspectives and is keen to keep developing. (GP6)* |
| **Macrosystem- Q5. If we think about exceptional care in terms of the interface between the GP practice and other healthcare services, such as hospital care- what does that look like to you?** | | | |
| **Access to specialty primary care services (Macro) (79%)** | Primary services are located in close proximity (55%) | - Develop primary care centres with primary care services located within the same building (33%) | *“With regards to physios and the likes, ideally you'd like the primary care team to be all under one roof, to work as a team and communicate together.” (GP60)* |
|  |  | - Ensure the practice is located near or has good links with other primary care services (33%) | *“…the bottom line is really good general practice situated within really good primary care teams, and I mean, there would be a proximal pharmacy and a proximal, you know, public health nurse service and audiology service and child psychology service…” (GP6)* |
|  | Primary care services and supports can be accessed in a timely manner (52%) | - Develop clear referral pathways to other primary care services (e.g., pharmacy, OT, public health nursing, and other GPs) that can be accessed in a reasonable amount of time (48%) | *“Accessible, so that it's something that they can access, be seen by another service, you know as quickly as possible, and again when they go to that other service, whether it's a pharmacy or physio or anywhere else at all, that again that the environment there also meets their needs.” (GP3)* |
|  |  | - Identify and provide good access to important community services, such as local social prescribing and ambulance services (15%) | *“…if you look at practices that are doing social prescribing or that have social prescribers within them, the resources they have identified are huge and that information is valuable to everybody but it has been hard won for that practice.” (GP10)* |
| **Continuity of care (Macro) (64%)** | Lines of communication are established and information is continuous (36%) | - Maintain accessible lines of two-way communication with other primary care providers, such as by email or phone (21%) | *“…the GP and public health nurse can chat about the care of the people in the area, and I think that's really great for delivering exceptional care as well.” (GP9)* |
|  |  | - Keep patients' records updated with new information and share relevant patient details with other providers involved in a patient's care (18%) | *“…just think an exceptional service would allow a way where by a locum doctor would have access, particularly when they have to make a diagnosis, or they’ve been called out in an emergency situation.” (P13)* |
|  | Services are integrated and managed continuously (33%) | - Optimise the use of other primary care services for patient care (e.g., directing patients to pharmacies for vaccinations) (15%) | *“Even down to the flu jab, that your local pharmacy could deal with the flu jab, rather than clogging up the local clinic.” (P5)* |
|  |  | - Collaborate with other primary care providers and integrated care teams in the community (e.g., diabetes integrated care team) (18%) | *“…if you are needing to access a physio, or in my case, you know, both alternative and like osteopaths or other parts, they can all work together respectfully.” (P3)* |
|  | Care is kept in the community (30%) | - Develop ambulatory care hubs and clinics in the community, such as COPD and diabetes clinics (18%) | *“…the whole plans for the enhanced community care centres where you have full complements of those teams there, respirator, diabetes and heart hearts team it’s called...if it follows the same templates as their diabetes services, it should work very well.” (PN4)* |
|  |  | - Provide access to diagnostics in the community, such as X-Rays and ultrasound facilities (18%) | *“…we're lucky here where we are because we have access to mobile diagnostics, which is an X-Ray and ultrasound facility, and you know, it's amazing for patients, especially who don't have their own transport that can’t go to [the city] for things like that.” (PM2)* |
|  | Providers have rapport and relational continuity is supported (27%) | - Make effort to get to know other providers and build relationships (24%) | *“…it's about really having good relationships with these other providers is essential. We certainly made a huge effort at the start of the pandemic to build our relationships with the pharmacies, like I went in to drop off prescriptions on daily basis, but that actually builds our personal relationships with those pharmacies far more than anything else.” (PM2)* |
|  |  | - Use relationships and informal lines of communication to get patients necessary care (e.g., personal phone-call, visiting the service) (9%) | *“What I mean by that is the physios, I can nip across the road and say ‘this is urgent, could you see’, and they will see because they know me and they kind of trust you, and you build up a relationship.” (GP4)* |
| **Integration in the healthcare system (Network) (91%)** | Lines of communication are established and information is continuous (76%) | - Develop and use direct and open lines of communication to secondary colleagues (e.g., to ask questions about referrals, get estimated time for referral) (39%) | *“If there are good channels of communication setup between primary care doctors and our secondary care colleagues, where we can ask questions or ask for opinions. So, again, not everybody that we refer actually needs to physically drive into an appointment and sit in an outpatients…sometimes we're looking for their opinion on results or a situation…” (GP2)* |
|  |  | - Send appropriate and accurate information back to primary care promptly, with an appropriate amount of detail and plan for care (39%) | *“…that they’d get whatever it is the treatment is that they require or investigation or whatever it is, and that the result or the outcome of the treatment or investigation is communicated to the referrer as promptly as possible.” (GP5)* |
|  |  | - Develop patient health record that can be accessed by all providers, and share relevant information promptly with secondary care (27%) | *“Essentially, like using information communication technology to link everything into your health records that can be accessed from, everyone can access it, you know every different provider, and saves data, reduce waste and more efficient, you know.” (P8)* |
|  | Access to specialists and diagnostics is timely (39%) | - Identify and maintain referral pathways with prompt access directly to secondary care specialists (27%) | *“It’s timely access and seeing patients who need to be seen with worrying cancer symptoms…And actually consultant review, not necessarily review by a junior member of staff…” (GP13)* |
|  |  | - Identify and maintain direct access scopes and referral pathways with prompt access to diagnostics (24%) | *“…access to diagnostics is a big one, you know, and so much of why I use the hospital for patients is not because they're desperately ill…it's that they need something done, they need a test, they need whatever it is…” (GP2)* |
|  | Primary care is core of an integrated system (27%) | - Integrate primary care better in the infrastructure to provide seamless links between services (15%) | *“Yeah it's good to feel like you're in a system that's kind of linked up, you know, where it's not like you feel like you're seeing the GP in one isolated kind of health environment or kind of a silo.” (P8)* |
|  |  | - Use primary care resources as central to healthcare, supported by Government policy (12%) | *“…we’re the hub a window or the repository of most of the information, so yeah it is important, when you're translating to people what happened in the hospital and explaining to them what's going on...” (GP10)* |
|  | Providers build relationships and have regular interfaces (21%) | - Take part in common meetings and Continuing Medical Education schemes where providers can work together and develop mutual understanding (15%) | *“I think it’s usually very helpful as well when secondary care colleagues participate in CME schemes, where they would give us talks and workshops and things like that, so that we can understand what's going on at the moment in their speciality…” (GP2)* |
|  |  | - Build rapport and develop good working relationships with secondary care providers (9%) | *“Hospital definitely needs a lot of work, so an ideal hospital is one where there has already established relationships between primary and secondary care, and it is nurtured.” (GP4)* |

| **Access to care (National) (73%)** | Provides timely access to care for all (39%) | - Provide timely access to care, particularly for public patients (21%) | *“…exceptional care outside of general practice in a timely fashion, a pathway that's accessible and available, and just a natural pathway.” (PM1)* |
| --- | --- | --- | --- |
|  |  | - Provide free universal healthcare for all people within the country (21%) | *“Obviously, if it was free to access and if it was just publicly available for everyone, I would think that would be from my perspective, it would be a better system where people could access it easier and you know money wouldn't be a barrier.” (P8)* |
|  | Services that are geographically accessible (24%) | - Ensure that rural areas have a sufficient number of the right services that can be easily accessed, including transport services (18%) | *“…there needs to be sufficient facilities, so that people can go to a doctor in their area and it's not closing down. I guess, it’s particularly important for rural settings, they need, people need access to the doctor.” (P9)* |
|  |  | - Ensure secondary care services are situated and available within a reasonable distance geographically (12%) | *“…that people who need access are able to get it within a reasonable time and also within a reasonable distance from their house.” (P10)* |
|  | Provides care pathways for patients with special and complex needs (24%) | - Accommodate access to services for patients with complex needs (15%) | *“Maybe taking the patient into account a little bit more from the point of view of helping them with access insofar as whether transport if that’s an issue or if mobility is an issue or...some form of service that can be provided within the home to a certain degree.” (GP5)* |
|  |  | - Provide accessible pathways to all specialty healthcare services (e.g., mental health services, transgender healthcare) (12%) | *“…there’s kind of adequate services that GP can refer to, you know, particularly with mental health.” (GP8)* |
| **Funding and resourcing (67%)** | Manages the GP workforce and recruitment (48%) | - Train and maintain a sufficient number of the right staff to support general practice (42%) | *“…increasing the number of GPs, I know they're increasing the number of GP trainings but actually if you had more GPs, if you had more practices, it might reduce demands on practice.” (GP60)* |
|  |  | - Create a good working environment and work-life balance for GP staff (e.g., ensuring adequate maternity leave, appropriate salary) (9%) | *“GPs, everybody salaried properly, nurses salaried properly, everyone salaried properly on proper HSE pensions, so that there’s job security, all that sort of stuff, you know, understandably some expectations.” (GP6)* |
|  |  | - Optimise the capacity of the workforce (e.g., move the limited number of advanced nurse practitioners around different practices) (6%) | *“…it can be worked out, allocate people, maybe a day here, a day there, have it set, ‘you're a day in this town, then you’re a day in this one’, and work it out properly.” (PN3)* |
|  | Provides adequate funding that is appropriately allocated (39%) | - Invest an appropriate proportion of the budget to the right areas of primary care and provide clarity around spending (36%) | *“We invest 3.7% of HSE budget in primary care versus NHS Scotland who put 10% into general practice, most of the UK is about 7- 8%. So, we are way below where we should be in terms of funding.” (GP1)* |
|  |  | - Provide appropriate reimbursement for services provided to public patients (6%) | *“…there’s adequate remuneration there for all of what general practice does.” (GP8)* |
|  | Provide incentives and supports to facilitate care delivery (27%) | - Provide grants and subsidies to help practices update their infrastructure and increase capacity (e.g., in rural or disadvantaged areas) (18%) | *“…before we used to have this extra money that you got for people who lived far away from the practice...it was kind of like a rural subsidy...” (GP9)* |
|  |  | - Develop incentives to encourage staff to achieve high-quality care (e.g., chronic disease management programme, supports for providing additional services in primary care) (12%) | *“It's not beyond the wit of the department of health to develop incentives to push people in the right direction, and that's what in fairness the Chronic Disease Management programme is.” (GP1)”* |
|  | Invests in developing primary care infrastructure (24%) | - Invest in practice buildings and providing more space in general practice (18%) | *“They could invest more in the practices, like we were talking about earlier with the ideal practice set up, a lot of practices are smaller… access issues, parking issues.” (GP60)* |
|  |  | - Invest in developing IT infrastructure and advanced equipment (e.g., advanced diagnostic services in general practice) (15%) | *“…if they were looking to invest more in GPs, GP practices, provide more like advanced equipment, maybe, you know, I’m not sure as to what that would be, so that we can take a bit of strain off hospitals.” (P2)* |
| **Support and guidance (National) (48%)** | Provides training and supports for staff in general practice (30%) | - Provide practical guidance to staff in GP, and develop national repositories of information about local services (18%) | *“Potentially like a database, or something where you could easily find referral pathways. There are many things that are coming more and more in to this century, this decade that the health care system in Ireland isn't prepared for…”(PM1)”* |
|  |  | - Provide standardised and tiered level education for all staff in general practice, including practice nurses and practice management (15%) | *“…to improve and standardize practice management across practices and having more support there. That's one of the [national GP college] requests in this current budget submission…they’re requesting support for project managers...” (PM2)”* |
|  | Engages general practice meaningfully in strategies and initiatives (24%) | - Involve all general practice staff at the front line meaningfully in policy making, including GPs, practice nurses and administrative staff (18%) | *“in terms of delivering exceptional care, like yes, we can do within the practice, but to do it at scale…we need primary care, we need secondary care, we need the HSE, we need academia, we need the voluntary sector and we need patient voices…” (GP1)* |
|  |  | - Consider general practice in the development of strategies and initiates, e.g., National Safety Policy (12%) | *“I think it’s really important that, you know, even like the care model in Ireland, that there is recognition that GP needs to be considered within strategies and within safety initiatives and all those things… (P1)”* |
|  | Provides accountability and quality oversight for general practice (18%) | - Provide quality oversight for general practice and develop system for accountability of goals, deadlines, and roles between care services (15%) | *“…a system for accountability and for like goals and deadlines, or I suppose meeting indicators and targets, all that, because I think it's just inefficiency.” (P8)* |
|  |  | - Develop appropriate quality indicators, and support and encourage staff to go for quality marks (6%) | *“...encourage practices to go for quality marks, put a system in place for them put you know, give them direction, how they might look at the different quality marks, the different indicators, different things like that.” (PN3)* |
|  | Allows general practice to maintain autonomy in providing care (12%) | - General practice care is structured around patient need, rather than politics (6%) | *“…your health service has got to be recognized not based upon votes, but based upon community need. (P13)”* |
|  |  | - Allow general practice staff autonomy to deliver care how they deem appropriate on a a local level (6%) | *“...everyone salaried properly on proper HSE pensions, so that there’s job security, all that sort of stuff, you know... you'd want the same autonomy the hospital consult has, you do not want to be told by the HSE what to do... (GP6)”* |

^a^Percentage of patients who reported a strategy at this category

^b^For the participant identifiers, ‘P’ denotes a patient, ‘GP’ denotes a general practitioner, ‘PN’ denotes a practice nurse, and ‘PM’ denotes a practice manager
